# Selectively Fluorinated Citronellol Analogues Support a Hydrogen Bonding Donor Interaction with the Human OR1A1 Olfactory Receptor

**DOI:** 10.1021/acs.orglett.2c01635

**Published:** 2022-06-10

**Authors:** Mengfan He, Weihong Liu, Chen Zhang, Yingjian Liu, Hanyi Zhuang, David O’Hagan

**Affiliations:** † School of Chemistry, University of St. Andrews, St. Andrews, KY16 9ST, U.K.; ‡ Intelligent Perception Lab, Hanwang Technology Co., Ltd., Beijing, 100193, China

## Abstract

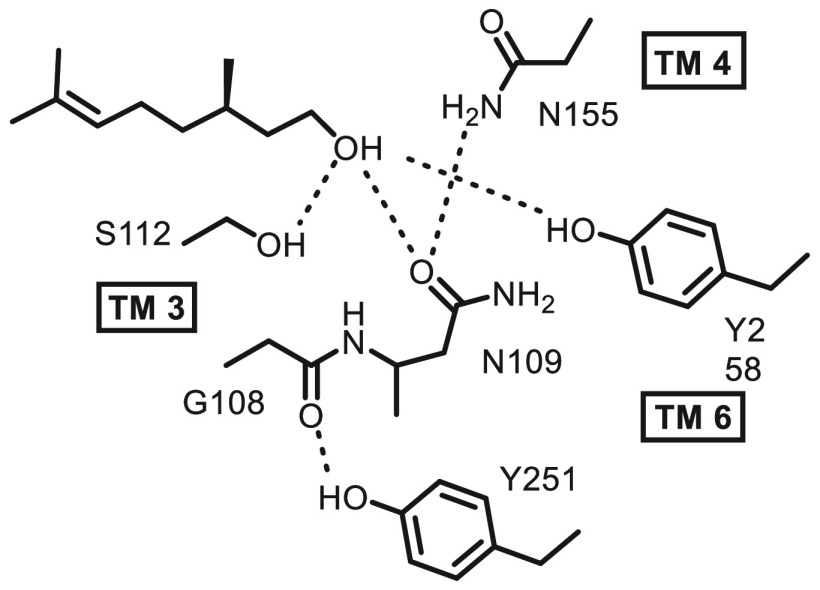

C-2 fluorinated and methylated stereoisomers of the fragrance citronellol **1** and its oxalate esters were prepared from (*R*)-pulegone **11** and explored as agonists of the human olfactory receptor OR1A1 and assayed also against site-specific mutants. There were clear isomer preferences and C-2 difluorination as in **18** led to the most active compound suggesting an important hydrogen bond donor role for citronellol **1**. C-2 methylation and the corresponding oxalate ester analogues were less active.

There is an ongoing interest in understanding how fragrance molecules act.^1^ Olfactory receptors fall into the class of G-protein coupled receptors which were initially cloned in 1991, and more were then identified as a consequence of the human genome sequencing projects.^2^*In vitro* expression of some of these genes combined with site-directed mutagenesis offers an approach to identifying key binding ligands within olfactory receptors, although structural studies of this class of transmembrane receptor have proven challenging.^3^ Cryo-electron microscopy has allowed structural elucidation of several olfactory receptors from higher organisms, although no human olfactory receptor structure has been resolved to date.^1a,4^ Collectively, mutagenic and structural strategies are providing data to develop more refined hypotheses regarding the nature of ligand binding sites.^4^ The emerging hypotheses indicate that ligands (fragrance molecules) bind to highly hydrophobic sites in the receptor that may accept a range of species^5^ although these receptors are somewhat less responsive to the subtle effects of stereochemistry and more so to overall shape.^6^

In this study we have selected the human olfactory receptor OR1A1^7^ to explore structure–activity relationships stemming from citronellol **1**. This is a broadly tuned receptor that will accept a range of molecular motifs as agonists.^3a,5b^ While it has been identified as a musk receptor, it is also triggered by small terpenes such as carvone and limonene, as well as citronellol **1** and citronellal.^3a,8^ Both enantiomers of citronellol trigger the OR1A1 receptor with moderate to good activity (∼80–90 *m*M EC_50_) and with no significant stereochemical discrimination between enantiomers.^8a^ The hydrogen bonding donor and acceptor ability of the alcohol OH group to active site amino acids is implicated as an important binding mechanism.^3a,8^ The enantiomers of citronellol are fragrant natural products which are extracted from a range of lemongrass plants. They are used both as a fragrance (rose oil) and as an insect repellent and may confer some health benefits.^9^ This study set out to explore the influence of selective fluorinations and also methylations on the agonist activity of citronellol with the OR1A1 receptor (Figure 1). Fluorine is the next smallest atom to hydrogen that forms a stable covalent bond to carbon; however, unlike hydrogen, it is highly electronegative and can be used to probe stereoelectronic over steric effects.^10^ We and others have developed an interest in exploring a role for selective fluorination of fragrance molecules to gain a deeper understanding of conformation and the nature of key interactions of small molecule fragrances to their receptors.^11^

**Figure 1:**
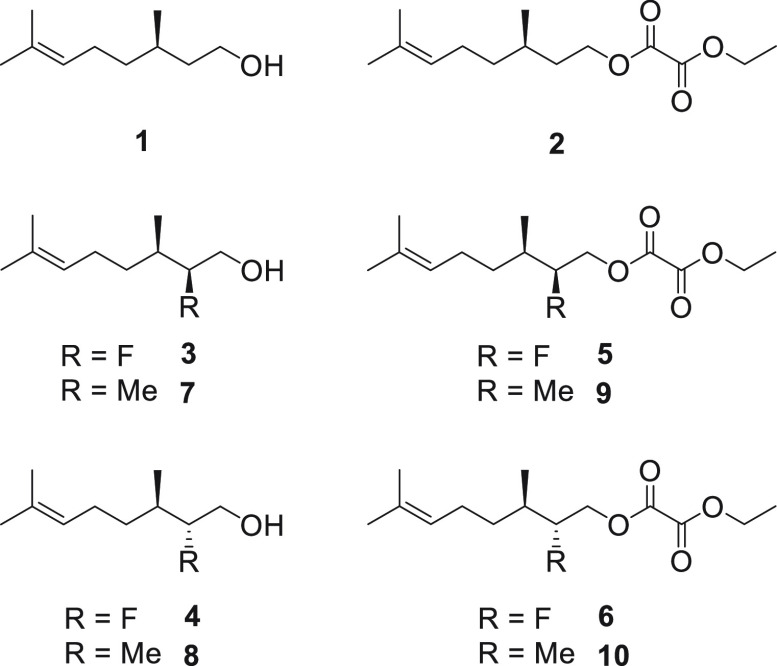
Citronellols (left) and citronellol oxalate esters (right).

Fluorine substitution should not have a significant steric effect; however, C-2 fluorination is anticipated to render the alcohol moiety of citronellol **1** a better hydrogen bonding donor due to the electronegativity of the fluorine further polarizing the alcohol hydrogen. On the other hand, C-2 methylation would have less of an electronic influence on the alcohol, but the resultant diastereoisomers may impact sterically. As an extension to the study of modified citronellols (fluorinated and methylated), we have also explored the corresponding ethyl oxalate esters. (*R*) Ethyl citronellyl oxalate **2** (ECO) has been described as having both a musk and also a “rose like” fragrance, and it has been developed for use in various fragrance products.^6,12^ However, ECO **2** is not a hydrogen bond donor and in that respect is distinct from citronellol **1**.

An overview of the synthesis routes to the various selectively fluorinated and methylated analogues of citronellol **1** and ECO **2** is illustrated in Scheme 1. All of the synthetic targets originated from (*R*)-pulegone **11** as the basis of establishing the stereointegrity of the C-3 methyl group of the citronellol skeleton. The routes also took advantage of the well-established ring opening protocol of pulegone to carboxylic acid **12**.^13^

**Scheme 1:**
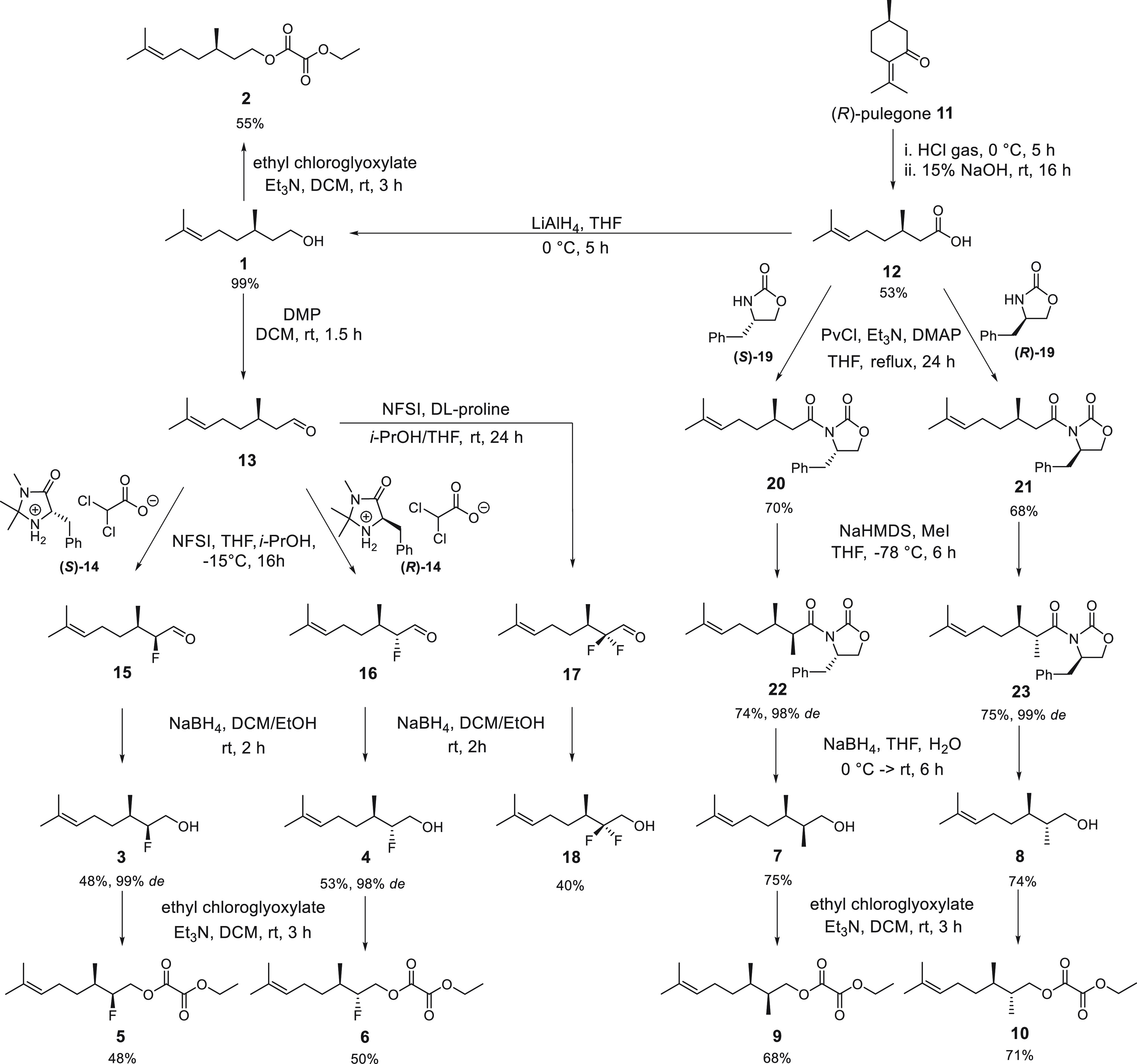
Synthetic Routes to Citronellol and Oxalate Ester Analogues from (*R*)-Pulegone **11**

For the fluorine series, carboxylic acid **12** was reduced to citronellol **1** with LiAlH_4_.^14^ A sample of citronellol was also progressed to the corresponding ECO **2** as a reference compound.^15^ For fluorination, citronellol was selectively oxidized to the corresponding aldehyde **13**.^16^ Application of a MacMillan α-fluorination protocol^17^ using the enantiomers of the imidazolidinone organo-catalyst (*S*)-**14** and (*R*)-**14**, followed by *in situ* reduction for the resultant α-fluoroaldehydes **15** and **16**, allowed alcohols **3** and **4** to be isolated, essentially as single stereoisomers. The persistence of the indigenous C-3 methyl group with its defined stereogenicity enabled a straightforward assessment of the diastereoselectivity (*de*) of these fluorination reactions by ^19^F{^1^H}-NMR, and they were consistently very high (∼98% *de*). In each case a sample of the resultant alcohol was also progressed to the corresponding ethyl oxalate esters **4** and **5** using previously described protocols.^15^

Preparation of the selectively methylated analogues used the Evans oxazolidinone, asymmetric alkylation approach.^18^ Carboxylic acid **12** was used to acylate the enantiomers of oxazolidinones **19**, to separately generate diastereoisomers **20** and **21**. Alkylation with methyl iodide then gave the corresponding diastereoisomers **22** and **23**, each in very high diastereoisomeric excess (∼99% *de*). Reductive removal of the auxiliary generated the desired alcohols **7** and **8**, and in each case a sample was also converted to the corresponding oxalate ester **9** or **10** respectively.

The 2,2-difluorocitronellol **18** was prepared to assess the nature of increased fluorination at C-2. This citronellol was also prepared from aldehyde **13** after a double fluorination using the MacMillan protocol^17^ and then *in situ* reduction of aldehyde **17** to generate **18**. Structural assignments were made with additional information from gCOSY, gHSQC, and gHMBC experiments. These citronellols and the oxalate esters **3**–**10** and **18**, were all evaluated as agonists of the human olfactory receptor OR1A1. As a first impression we found the three fluoro derivatives all to have a similar “alcoholic” fragrance to citronellol, whereas the Me derivatives far less so.

**Olfactory Receptor Activity Assays.** In order to assess the responses of the various citronellol and oxalate ester derivatives, we employed a HEK293T cell-based heterologous expression system for the human olfactory receptor OR1A1.^19,20^ The HEK293T cells enable an effective functional expression of most mammalian olfactory receptors, as they also express accessory proteins that are native to the olfactory epithelium where the olfactory receptors are mainly expressed. A downstream luciferase-based reporter assay then allows the response of each OR1A1 agonist/ligand to be recorded. (See Supporting Information for full experimental procedures.)

The results of the citronellol analogue assays against the OR1A1 receptor are summarized in Figure 2a. The most striking outcome is that the (2*S*, 3*R*)-monofluoro- stereoisomer **3** has the strongest (taken as 100%) response with the OR1A1 receptor. The (2*R*, 3*R*)-monofluoro- stereoisomer **4** also displays a significantly increased response relative to citronellol **1** however not as strong as **3** which is indicative of a stereoelectronic influence of the fluorine, perhaps securing diastereoisomeric conformations due to intramolecular CF···HO bonding (Figure 2b). Interestingly the 2,2-difluorocitronellol **18** displays the highest potency of all isomers tested, although it only reaches up to 50% of the efficacy of stereoisomer **3** (Table S1). These C-2 fluorinated analogues are all more efficacious than citronellol **1** itself, which is a poor agonist in this assay. The C-2 methylated citronellol stereoisomer **7** is not active at all, and **8** is similar to citronellol **1** perhaps indicating an adverse steric interaction between the C-2-Me and the OH in **7**, which is inverted in **8** (see Figure 2b). The outcomes of the ethyl oxalate ester (ECOs) assays are summarized in Figure S1. In general, these oxalate esters are all weaker agonists relative to the citronellols as a group, and the C-2 methyl analogues **9** and **10** have similar activity to reference compound **2**. Again, the monofluoro stereoisomers, **5** and **6**, are the most active compounds of the series, more so than ECO **2**, indicating a positive fluorine effect, although the origin of the effect is not clear.

**Figure 2:**
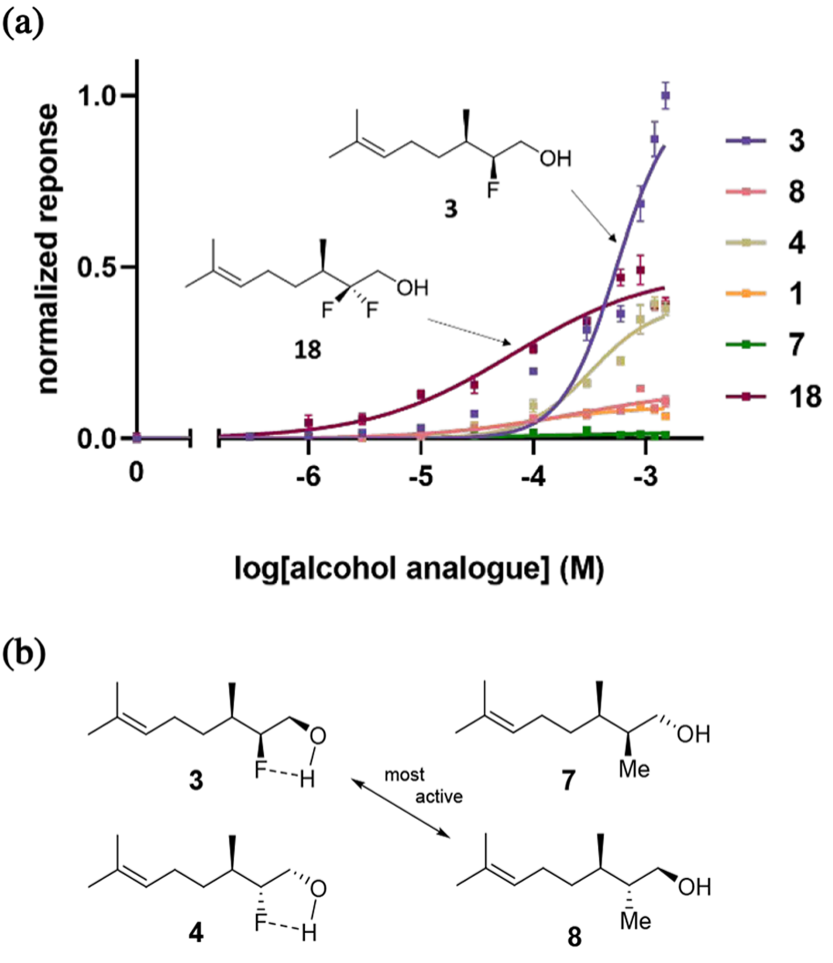
(a) Dose–response curves for citronellols and (b) rationalization of citronellol binding conformation against the human olfactory receptor OR1A1.

**OR1A1 Mutant Studies.** In order to explore further the importance of a hydrogen bonding interaction between the citronellol hydroxyl group and the receptor, the most potent and the most efficacious agonists (**3** and **18**) were assayed against site-specific mutants of the OR1A1 receptor, removing amino acid side groups that were previously implicated in hydrogen bonding to citronellol and other OR1A1 ligands.^8a^ In total, five mutants were explored. These were N109A, S112A, N115A, Y251F, and Y258F, and each mutant was challenged with the monofluoro (2*S*, 3*R*)-**3** and the 2,2-difluoro **18** analogues of citronellol. The data are summarized in Figure 3a and Figure 3b, respectively. In each case three of the mutants (N109A, S112A, and Y258F) displayed very significantly reduced activity relative to the wild type OR1A1 receptor, indicating the importance of these specific residues for successful binding. The Asp155 had previously been implicated in hydrogen bonding to the hydroxyl group of citronellol;^8a^ however, mutation of this residue to alanine gave a fully competent receptor, suggesting that it is not involved directly in bonding the ligand. The OR1A1 mutant where Tyr-251 was switched to Phe-251 had a partial effect on the agonist ability of **3** and a much more deleterious influence on the activity of **18**, suggesting a hydrogen bonding role for the Tyr-251 OH group.

**Figure 3:**
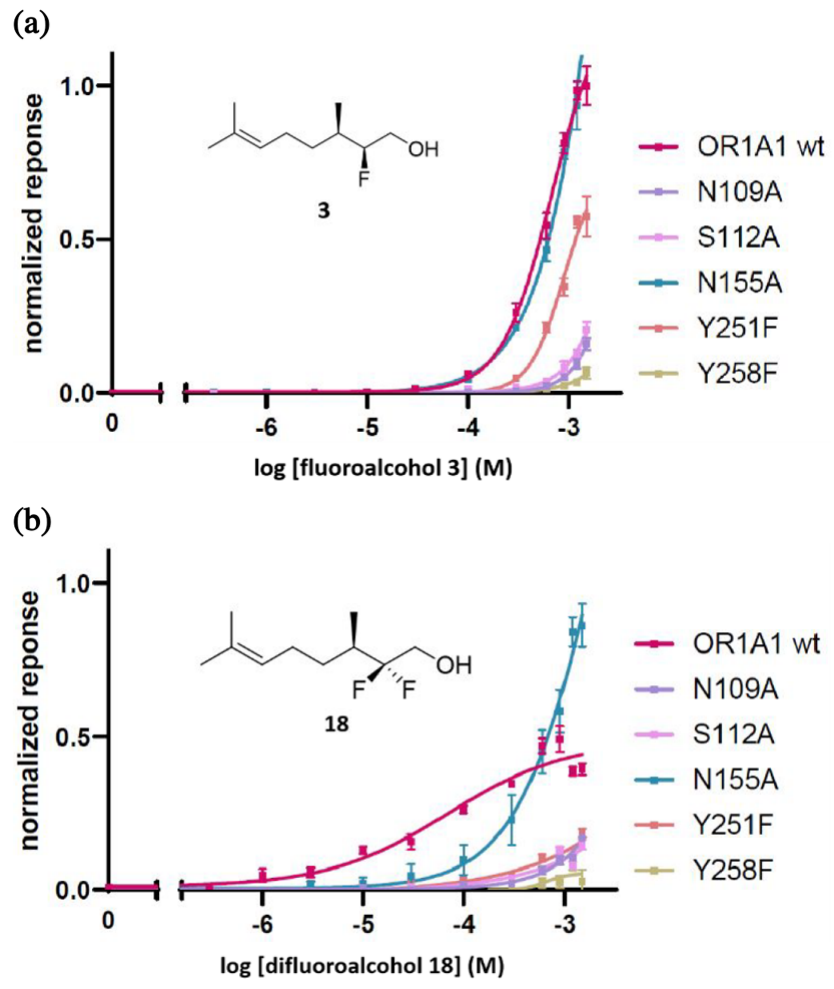
Dose–response curves for (a) **3** and (b) **18** against site-specific mutants of the OR1A1 receptor.

**Discussion.** Agonist efficacy increases for the C-2 monofluorinated citronellols **3** and **4**, and this further increases with the difluorinated analogue **18** suggesting an important hydrogen bonding donor role of citronellol. For the oxalate esters the C-2 fluoro- stereoisomers **9** and **10** also showed improved activity over the parent oxalate **2**. In contrast selective C-2 methylation exhibits no improved activity of the citronellol or the oxalate esters.

A recent study of Linclau et al.^21^ explored the hydrogen bonding donor ability of selectively fluorinated alcohols. Log P values do not suffice as a measure of H-bonding ability because fluorine introduces polar effects independent of the isolated hydrogen bonding interaction, so an FT-IR approach was taken to examine the strength of the hydrogen bonding component only, across a series of conformationally biased fluorinated *tert*-butyl cyclohexanols alcohols. A summary of outcomes is shown in Figure 4.

**Figure 4:**
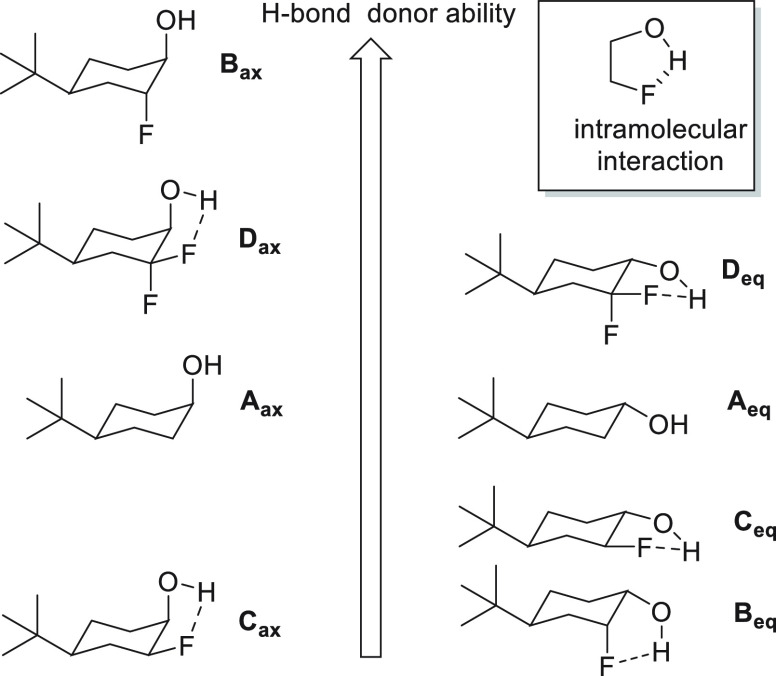
Relative H-bonding donor ability of vicinal fluoro-alcohols as described by Linclau et al.^21b^

For example, when fluorine is placed vicinal to either an axial (*ax*) or an equatorial (*eq*) −OH, the hydrogen bonding donor ability (acidity) is stereochemically dependent. The *ax*/*ax* isomer **B**_***ax***_ is a better H-bond donor than the parent alcohol **A**_***ax***_, whereas the *ax*/*eq* isomer **C**_***ax***_ is less good. This is because isomer **C**_***ax***_ accommodates an intramolecular hydrogen bond which attenuates intermolecular H-bonding donor ability. In the difluoro case **D**_***ax***_, the second fluorine improves the H-bond acidity, but not to the level of the *ax*/*ax* isomer, as there is still capacity for an intramolecular hydrogen bond to the equatorial fluorine. For isomers **B**_***eq***_ and **C**_***eq***_ in the equatorial −OH series, both isomers are weaker hydrogen bonding donors relative to the alcohol **A**_***eq***_ again due to intramolecular hydrogen bonding; however, the second fluorine in **D**_***eq***_ increases the hydrogen bonding donor ability above the parent alcohol. The monofluorinated citronellols **3** and **4** studied here will have increased conformational flexibility^21a^ and will be less able to accommodate intramolecular hydrogen bonding due to their increased flexibility; therefore, the inductive influence of fluorine will be more significant. This results in isomers **3** and **4** being better hydrogen bonding donors than citronellol **1** and analogue **18** again.

These observations support Schmiedeberg et al.,^8a^ who recognized the importance of citronellol **1** as a hydrogen bonding donor on the receptor. Site-specific mutations of the receptor add further support. We find that mutations of the Ser-112 and Arg-109 residues result in very poor agonist responses and the mutation of Tyr-258 to Phe-258 almost abolishes agonist activity with **3** and **18**. In a previous study^3a^ we concluded that Tyr-258 was important in forming a hydrogen bonding interaction to muscone on this receptor, and the data indicate a role for citronellol too (Figure 5).

**Figure 5:**
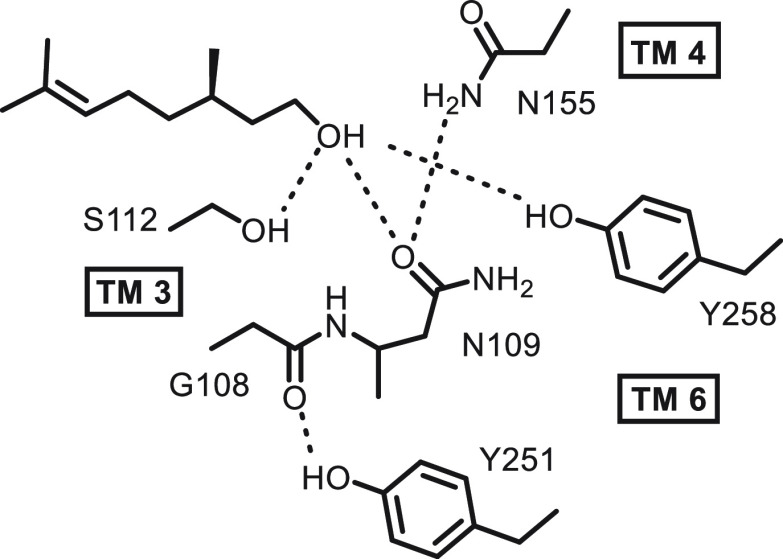
Putative hydrogen bonding interactions of citronellol in the OR1A1 receptor developed from Schmiedeberg.^8a^

It is notable that the methyl analogues **7**/**8** or **9**/**10** do not significantly change activity in the citronellol or oxalate ester series and that the electronic effect displayed by fluorine is much more significant.

In conclusion, we present results in which fluorine has been used as a tool to explore the importance of hydrogen bonding in this small molecule–receptor interaction. The results are consistent with developing hypotheses for the OR1A1 receptor, but the approach could be applied more widely.

## Supporting Information Available

Synthetic procedures, characterization, olfactory receptor activity assays, and spectral data (PDF)
