# Supplementary material for: Selectively Fluorinated Citronellol Analogues Support a Hydrogen Bonding Donor Interaction with the Human OR1A1 Olfactory Receptor
Source: Org Lett. 2022 Jun 10;24(24):4415–20. doi: 10.1021/acs.orglett.2c01635 (PMC9237825; doi:10.1021/acs.orglett.2c01635)
Supplement: Supplementary file 1 — ol2c01635_si_001.pdf [file ol2c01635_si_001.pdf]

SUPPLEMENTARY INFORMATION

Selectively Fluorinated Citronellol Analogues Support a Hydrogen Bonding Donor Interaction with the Human OR1A1 Olfactory Receptor

Mengfan He,[a] Weihong Liu,[b] Chen Zhang,[b] Yingjian Liu,[b] Hanyi Zhuang,*[b] and David O'Hagan*[a]

[a] *School of Chemistry, University of St Andrews, St Andrews, KY16 9ST, UK.*
[b] *Intelligent Perception Lab, Hanwang Technology Co., Ltd., Beijing, 100193, China.*

* *E-mail: do1@st-andrews.ac.uk*

Table of Contents

General 2

Synthesis protocols and NMR data 3

NMR Spectra 17

Additional experimental procedures 47

Figure S1 Oxalate ester dose response curves against the human olfactory receptor OR1A1. 48

References 49

General

NMR Spectra were recorded on Bruker AVIII 500, AVIII-HD 500 or AVIII-HD 700 spectrometer. NMR analyses were carried out at room temperature in indicated deuterated solvents unless otherwise noted. Chemical shift data are reported as δ in units of ppm relative to respective deuterated NMR solvent. Coupling constant J was reported in Hz. ^{1}H, ^{13}C, ^{19}F NMR spectra were recorded at 470 MHz with and without ^{1}H decoupling, relative to CCl_3F ($\delta F = 0.00$ ppm). Structural assignments were made with additional information from gCOSY, gHSQC, and gHMBC experiments in conjunction with ^{1}H, ^{13}C, and ^{19}F NMR data. Multiplicities are indicated by: s for singlet, d for doublet, t for triplet, q for quartet, p for septet and m for multiplet and br. for the broad brand.

All reactions were carried out under an argon atmosphere with standard Schlenk techniques unless otherwise specified. The reaction glassware was flame dried or oven dried and cooled under vacuum. Commercially available chemicals were purchased from Acros, Alfa Aeser, Fisher Scientific, Fluorochem, Sigma Aldrich, Strem Chemicals, and TCI (UK) and used as received unless otherwise stated.

DCM and THF were dried and deoxygenated using an MBraun SPS-800 solvent system. Room temperature refers to the temperature range 15-25 ^{o}C. *In vacuo* refer to the use of rotary evaporator with membrane pump at 30-50 mbar Analytical thin-layer chromatography was carried out on aluminium backed Merck TLC silica gel 60 F254 plates. These plates were visualised using UV light at 254 nm wavelength, dyed by potassium permanganate or phosphomolybdic acid followed by air dryer heating. Flash column chromatography was performed with Sigma-Aldrich silica gel, 60 Å pore size and 230-400 mesh, 40-63 μm particle size under 5 psi compressed air. High resolution mass spectra were recorded on a Thermo Scientific Exactive orbitrap mass spectrometer by the University of St Andrews, UK.

Synthesis protocols and NMR data

(*R*)-3,7-Dimethyloct-6-enoic acid ((*R*)-citronellic acid, 12)

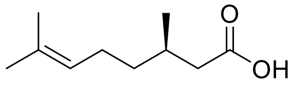

This method is modified from the procedure of Plesek.[1] (*R*)-Pulegone **11** (15.3 g, 0.1 mol) was cooled in a 50 mL two neck flask at 0 °C. Excess HCl (13.32 g, V(37% HCl aq) = 30 mL, 0.365 mL) was dropped onto concentrated H_2SO_4 to generate HCl gas. The gas was dried by passing through concentrated H_2SO_4 and anhydrous $CaCl_2$, and then bubbled through **11** for 5 h. After the addition was complete, the reaction could be followed by TLC (hexane : ethyl acetate = 6 : 1) to completion. The solution of the two isomers could be used directly without isolation.

The mixture above was added to 5% NaOH solution (15 g in 300 mL water, 0.375 mol) in a 500 mL flask at rt and was stirred vigorously overnight. The mixture was washed with Et_2O (3 x 50 mL).The aqueous phase was acidified with 37% HCl to pH 3 and an immediate yellow oil formed on the surface. This mixture was extracted into Et_2O (3 x 60 mL). The combined organics were dried over anhydrous Na_2SO_4 and concentrated under reduced pressure to give the product as a yellow oil **12** (8.95 g, 53%). ^{1}H NMR (400 MHz, Chloroform-*d*) δ 5.09 (tdq, *J* = 7.2, 2.9, 1.4 Hz, 1H), 2.37 (dd, *J* = 14.9, 5.8 Hz, 1H), 2.15 (dd, *J* = 15.0, 8.3 Hz, 1H), 2.08 – 1.89 (m, 3H), 1.68 (q, *J* = 1.3 Hz, 3H), 1.60 (d, *J* = 1.3 Hz, 3H), 1.38 (dddd, *J* = 13.4, 9.3, 6.6, 5.8 Hz, 1H), 1.31 – 1.17 (m, 1H), 0.98 (d, *J* = 6.6 Hz, 3H). $[\alpha]^{20}_D$ = +9.12° (c = 0.1, $CHCl_3$); Lit.: $[\alpha]^{22}_D$ = +8.20° (neat)[2]

(*R*)-3,7-Dimethyloct-6-en-1-ol ((*R*)-citronellol, 1)

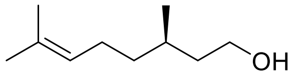

A solution of **12** (8.95 g, 0.053 mol) in dry THF (45 mL) was gradually added to a suspension of $LiAlH_4$ solution (2.5 g, 33 mL of 2M, 0.066 mol) in a 250 mL. The flask that was kept under N_2 at 0 °C with stirring for 5 h. Water (5 mL) was added cautiously and the whole was diluted with 30 mL ethyl acetate and then filtered with the aid of celite with suction. The filtercake was washed with ethyl acetate and the filtrate was dried over anhydrous Na_2SO_4 and concentrated under reduced pressure to give the product as a light yellow oil **1** (8 g, 99%). ^1H NMR (400 MHz, Chloroform-*d*) δ 5.09 (tdp, *J* = 7.4, 3.1, 1.5 Hz, 1H), 3.75 – 3.61 (m, 2H), 2.04 – 1.88 (m, 2H), 1.68 (q, *J* = 1.3 Hz, 3H), 1.67 – 1.51 (m, 5H), 1.43 – 1.33 (m, 2H), 1.17 (dddd, *J* = 13.4, 9.4, 7.6, 6.0 Hz, 1H), 0.90 (d, *J* = 6.6 Hz, 3H). $[\alpha]^{20}_D$ = +4.13° (c = 0.1, $CHCl_3$); Lit.: $[\alpha]^{20}_D$ = +4.57° (c = 2.21, $CHCl_3$)[3]

(*R*)-3,7-Dimethyloct-6-enal ((*R*)-citronellal, 13)

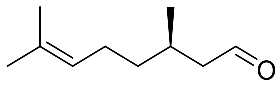

DMP (3.257 g, 7.68 mmol) was added to a solution of **7** (800 mg, 5.12 mmol) in dry DCM (25 mL) at 0 °C and the reaction was warmed to rt. After 1.5 h of stirring, the reaction was quenched with saturated 20% $Na_2S_2O_3$ solution (20 mL) and $NaHCO_3$ solution (30 mL) for 30 min. The aqueous layer was extracted into ethyl acetate (3 x 50 mL) and brine (50 mL), and the combined organics was dried over anhydrous Na_2SO_4 and concentrated under reduced pressure. The product was purified over silica gel (hexane : ethyl acetate = 30 : 1 to 20 : 1 to 15 : 1 and TLC hexane : ethyl acetate = 4 : 1) to give the product as a yellow oil **13** (234.0 mg, 44%). ^1H NMR (300 MHz, Chloroform-*d*) δ 9.75 (t, *J* = 2.4 Hz, 1H), 5.08 (ddp, *J* = 7.0, 5.8, 1.4 Hz, 1H), 2.46 – 2.16 (m, 2H), 2.14 – 1.92 (m, 3H), 1.68 (d, *J* = 1.4 Hz, 3H), 1.60 (d, *J* = 1.4 Hz, 3H), 1.44 – 1.28 (m, 2H), 0.97 (d, *J* = 6.6 Hz, 3H). $[\alpha]^{20}_D$ = +14.75° (c = 0.1, $CHCl_3$); Lit.: $[\alpha]^{20}_D$ = +14.1° (c = 0.1, $CHCl_3$).[3]

Synthesis of MacMillan catalyst precursor (General procedure A)

A solution of methylamine in ethanol (33%) (7 eq.) was added to a round bottom flask equipped with a magnetic stir bar and charged with ethyl 2-amino-3-phenylpropionate hydrochloride salt (**14a**, 1 eq.). The mixture was stirred for 48 h at rt and was then concentrated under reduced pressure. The resulting oil was dissolved in chloroform (50 mL), washed with saturated K_2CO_3 (50 mL) and extracted into chloroform (3 x 50 mL). The organics were dried over anhydrous Na_2SO_4, filtered and concentrated under reduced pressure. This material was used without further purification.

A two-neck round bottom flask with a Soxhlet (charged with 4 Å MS) and a reflux condenser was charged with 2-amino-N-methyl-3-phenylpropanamide (**14b**, 0.33 eq.) and *p*-toluenesulfonic acid (0.1 eq.). Anhydrous ethanol (80 mL) and anhydrous acetone (15 mL) were added and the mixture was stirred for 6 h under reflux (120 °C). The reaction was cooled to rt and concentrated under reduced pressure. The product was purified over silica gel to give **14c**. **14c** was then 1:1 mixed with dichloroacetic acid (DCA) and recrystalised to give **14**.

(*S*)-5-Benzyl-2,2,3-trimethylimidazolidin-4-one ((*S*)-14c)

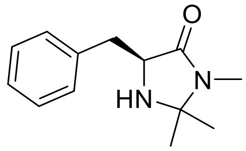

Imidazolidin-4-one **(*S*)-14c** was prepared according to General Procedure A from **(*S*)-14a** (2.5 g, 11 mmol), using methylamine in ethanol (33%) (94.66 mL, 77 mmol) and *p*-toluenesulfonic acid (10%, 78 mg, 1.1 mmol). Purification over silica gel (ethyl acetate : Acetone = 1 : 1 and TLC ethyl acetate : Acetone = 1 : 1) gave the product as a yellow oil (3.0 g, 98%). ^{1}H NMR (400 MHz, Chloroform-*d*) δ 7.36 – 7.15 (m, 6H), 3.85 – 3.74 (m, 1H), 3.18 – 2.95 (m, 2H), 2.74 (d, *J* = 0.7 Hz, 3H), 1.79 (s, 1H), 1.25 (s, 3H), 1.15 (s, 3H). Data is in agreement with that reported in the literature.[4,5]

(*R*)-5-Benzyl-2,2,3-trimethylimidazolidin-4-one (*R*-14c)

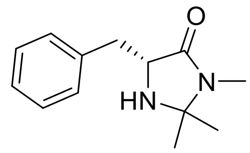

Imidazolidin-4-one **(*R*)-14c** was prepared according to General Procedure A from **(*R*)-14a** (2.5 g, 11 mmol), using methylamine in ethanol (33%) (94.66 mL, 77 mmol) and *p*-toluenesulfonic acid (10%, 78 mg, 1.1 mmol). Purification over silica gel (ethyl acetate : Acetone = 1 : 1 and TLC ethyl acetate : Acetone = 1 : 1) gave the product as a yellow oil (2.8 g, 98%). ^{1}H NMR (500 MHz, Chloroform-*d*) δ 7.36 – 7.07 (m, 6H), 3.78 (dd, *J* = 6.9, 4.5 Hz, 1H), 3.19 – 2.94 (m, 2H), 2.74 (s, 3H), 1.73 (s, 3H), 1.25 (s, 1H), 1.14 (s, 3H). Data is in agreement with that reported in the literature. [4,5]

Synthesis of fluorinated (*R*)-citronellol (General procedure B)

N-Fluorobenzenesulfonimide (NFSI, 5 eq.) and **14** (20 mol%) were added to a solution of **13** in THF(13.5 mL) and isopropanol (1.5 mL) at -15 °C and the mixture was stirred for 16 h. The reaction was diluted with Et_2O (10 mL) and filtered through a pad of Davisil® Silica Gel, eluting with Et_2O. Me_2S (10 mL) was added forming a white precipitate. The resulting mixture was washed with saturated $NaHCO_3$ solution (3 x 150 mL) and brine (1 x 150 mL) and dried over anhydrous Na_2SO4, filtered and concentrated under reduced pressure. The resulting oil was dissolved in DCM (12 mL) and ethanol (8 mL) and then $NaBH_4$ (2.5 eq.) was added at 0 °C and the reaction was left to warm to rt with stirring. After 1 h, saturated NH_4Cl solution (150 mL) was added at 0 °C. The mixture was warmed to rt and stirred vigorously 1 h. The cloudy suspension was allowed to separate and 75 mL of DCM was added. The solution was extracted into DCM (3 x 80 mL) and the combined organics were washed with saturated $NaHCO_3$ solution (3 x 100 mL) and brine (1 x 150 mL) and dried over anhydrous Na_2SO_4. The product was concentrated under reduced pressure and purified over silica gel.

(2*S*,3*R*)-2-Fluoro-3,7-dimethyloct-6-en-1-ol (3)

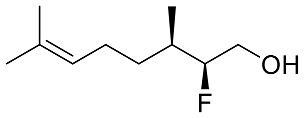

Alcohol **3** was prepared according to General Procedure B from **13** (210 mg, 1.36 mmol), NFSI (2.15 g, 6.8 mmol) and **(*S*)-14** (94.55 mg, 0.27 mmol) and was purified over silica gel (hexane : ethyl acetate = 40 : 1 to 35 : 1 to 25 : 1 to 15 : 1 to 10 : 1 and TLC hexane : ethyl acetate = 4 : 1) to give the product as a colourless liquid (113.9 mg, 48 %). ^{1}H NMR (300 MHz, Chloroform-*d*) δ 5.07 (ddp, *J* = 7.1, 5.7, 1.4 Hz, 1H), 4.55 – 4.29 (m, 1H), 3.92 – 3.56 (m, 2H), 2.04 (pt, *J* = 14.5, 7.9 Hz, 2H), 1.78 (dddd, *J* = 13.8, 8.7, 7.0, 3.5 Hz, 1H), 1.68 (q, *J* = 1.3 Hz, 3H), 1.60 (d, *J* = 1.4 Hz, 3H), 1.54 – 1.39 (m, 1H), 1.21 (dtd, *J* = 13.3, 8.9, 6.0 Hz, 1H), 0.96 (dd, *J* = 6.8, 1.1 Hz, 3H). ^{13}C NMR (126 MHz, Chloroform-*d*) δ 132.1, 124.1, 98.0 (d, *J* = 170.6 Hz), 63.7 (d, *J* = 22.3 Hz), 34.1 (d, *J* = 19.1 Hz), 32.7 (d, *J* = 4.0 Hz), 25.9, 25.4, 17.8, 14.3 (d, *J* = 6.4 Hz). ^{19}F{^{1}H}NMR (282 MHz, Chloroform-*d*) δ -197.6. IR (neat): 1221, 1508, 3318 cm^{-1}. HRMS (ES^{+}) *m/z*: [M + Na]$^{+}$ Calculated for $C_{10}H_{19}OFNa$ 197.1318; Found 197.1310. $[\alpha]^{20}_{D}$ = +12.67° (c = 0.1, $CHCl_3$).

(2*R*,3*R*)-2-Fluoro-3,7-dimethyloct-6-en-1-ol (4)

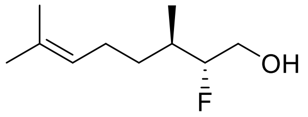

Alcohol **4** was prepared according to General Procedure B from **13** (500 mg, 3.24 mmol), NFSI (5.11 g, 16.2 mmol) and **(*R*)-14** (225.1 mg, 0.65 mmol) and purified over silica gel (hexane : ethyl acetate = 40 : 1 to 35 : 1 to 25 : 1 to 15 : 1 to 10 : 1 and TLC hexane : ethyl acetate = 4 : 1) to give the product as a colourless oil (299.4 mg, 53 %). ^{1}H NMR (500 MHz, Chloroform-*d*) δ 5.09 (tp, *J* = 7.0, 1.5 Hz, 1H), 4.34 (dddd, *J* = 49.2, 7.3, 5.5, 3.9 Hz, 1H), 3.80 – 3.70 (m, 2H), 2.13 – 1.90 (m, 2H), 1.86 (dddd, *J* = 13.3, 6.7, 4.0, 2.1 Hz, 1H), 1.82 (s, 1H), 1.68 (q, *J* = 1.4 Hz, 3H), 1.60 (d, *J* = 1.3 Hz, 3H), 1.57 (ddd, *J* = 10.3, 6.9, 3.8 Hz, 1H), 1.24 – 1.16 (m, 2H), 0.91 (d, *J* = 6.9 Hz, 3H). ^{13}C NMR (75 MHz, Chloroform-*d*) δ 132.0, 124.2, 98.5 (d, *J* = 170.2 Hz), 63.4 (d, *J* = 22.1 Hz), 34.0 (d, *J* = 18.8 Hz), 32.1 (d, *J* = 5.4 Hz), 25.9, 25.3, 17.8, 14.9 (d, *J*

= 6.6 Hz). $^{19}F\{^1H\}$NMR (282 MHz, Chloroform-*d*) δ -192.1. IR (neat): 1043, 1083, 2924 cm^{-1}. HRMS (ES^+) *m/z*: $[M + Na]^+$ Calculated for $C_{10}H_{19}OFNa$ 197.1318; Found 197.1308. $[\alpha]^{20}_D$ = +13.90° (c = 0.05, $CHCl_3$).

(*R*)-2,2-Difluoro-3,7-dimethyloct-6-en-1-ol (18)

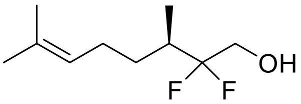

A solution of D,L-proline (92.1 mg, 0.8 mmol) and NFSI (1.26 g, 4 mmol) in 10% *i*-PrOH/THF (2 mL/18 mL) was stirred at rt and treated with **13** (308.5 mg, 2 mmol). The reaction mixture was stirred at this temperature for 24 h, and then cooled to -78 °C, diluted with 10 mL Et_2O and filtered through a pad of Davisil® Silica Gel, eluting with Et_2O. Me_2S (10 mL) was added, washed with saturated $NaHCO_3$ (3 x 20 mL) and brine (1 x 50 mL) and then dried over anhydrous Na_2SO_4, filtered and concentrated under reduced pressure. The resulting oil was dissolved in DCM (12 mL) and ethanol (8 mL) and $NaBH_4$ (189.2 mg, 5 mmol) was added at 0 °C and the reaction warmed to rt. After 1 h, saturated NH_4Cl solution (50 mL) was added at 0 °C. The mixture was warmed to rt and stirred vigorously 1 h. The cloudy suspension was allowed to separate and 75 mL of DCM was added. The solution was extracted with DCM (3 x 80 mL) and the combined organics washed with saturated $NaHCO_3$ (3 x 100 mL) and brine (1 x 150 mL) and dried over anhydrous Na_2SO_4 and concentrated under reduced pressure and purified over silica gel (hexane : ethyl acetate = 50 : 1 to 45 : 1 to 40 : 1 to 30 : 1 to 20 : 1 and TLC hexane : ethyl acetate = 4 : 1) to give the product as a colourless oil **18** (153.8 mg, 40%). ^1H NMR (300 MHz, Chloroform-*d*) δ 5.08 (ddp, *J* = 8.1, 5.6, 1.4 Hz, 1H), 3.87 – 3.69 (m, 2H), 2.18 – 2.02 (m, 2H), 2.02 – 1.90 (m, 2H), 1.68 (p, *J* = 1.6 Hz, 3H), 1.67 – 1.62 (m, 1H), 1.61 (d, *J* = 1.3 Hz, 3H), 1.26 (dddd, *J* = 13.3, 10.0, 9.1, 5.3 Hz, 1H), 1.03 (d, *J* = 7.0 Hz, 3H). ^{13}C NMR (126 MHz, Chloroform-*d*) δ 132.4, 123.8, 62.9 (t, *J* = 31.3 Hz), 36.3 (t, *J* = 22.4 Hz), 29.5 (t, *J* = 4.0 Hz), 25.9, 25.5, 17.8, 12.6 (t, *J* = 5.1 Hz). ^{19}F NMR (282 MHz, Chloroform-*d*) δ -114.9 (dd). IR (neat): 906, 1070

cm^{-1}. HRMS (ES$^+$) *m/z*: [M + Na]$^+$ Calculated for $C_{10}H_{18}OF_2Na_2$ 238.1121; Found 238.1107. $[\alpha]^{20}_D$ = +18.45° (c = 0.05, $CHCl_3$)

Synthesis of oxazolidinone (General procedure C)

To a solution of **12** (1 eq.), pivaloyl chloride (PvCl, 1.5 eq.) in dry THF (10 mL) was added Et_3N (2 eq.) at -78 ºC. The resulting mixture was then brought to 0 ºC and stirred for 1h. In a separate flask, 4-dimethylaminopyridine (DMAP, 0.5 eq.), Et_3N (3 eq.) and 4-benzyl-2-oxazolidinone **19** (1 eq.), were dissolved in dry THF (5 mL), and the solution was added dropwise to the reaction flask at 0 ºC. The resulting mixture was stirred under reflux conditions for 24 h. After cooling to rt, the reaction mixture was quenched with aqueous HCl (1 M 16.2 mL, 16.2 mmol). The aqueous layer was extracted with ethyl acetate (3 x 60 mL), and the organic layer was washed by brine (30 mL), dried over anhydrous Na_2SO_4, filtered and concentrated under reduced pressure. The product was purified over silica gel.

(*S*)-4-Benzyl-3-((*R*)-3,7-dimethyloct-6-enoyl)oxazolidin-2-one (20)

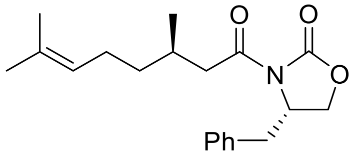

Oxazolidinone **20** was prepared according to General Procedure C from **12** (1.0 g, 5.874 mmol), PvCl (1.06 g, 8.81 mmol), Et_3N (4.1 mL, 29.37 mmol), DMAP (359.2 mg, 2.94 mmol) and **(*S*)-19** (1.04 g, 5.874 mmol) and purified over silica gel (hexane : ethyl acetate = 50/1 to 40/1 to 4/1 and TLC hexane : ethyl acetate = 4 : 1) to give the product as a yellow oil (1.4 g, 70%). ^{1}H NMR (300 MHz, Chloroform-*d*) δ 7.40 – 7.17 (m, 5H), 5.10 (tp, *J* = 7.1, 1.4 Hz, 1H), 4.68 (ddt, *J* = 10.2, 6.9, 3.4 Hz, 1H), 4.24 – 4.07 (m, 2H), 3.32 (dd, *J* = 13.3, 3.3 Hz, 1H), 2.87 (dd, *J* = 6.9, 2.0 Hz, 2H), 2.75 (dd, *J* = 13.3, 9.7 Hz, 1H), 2.22 – 1.89 (m, 3H), 1.68 (q, *J* = 1.3 Hz, 3H), 1.61 (d, *J* = 1.4 Hz, 3H), 1.53 – 1.36 (m, 1H), 1.36 – 1.18 (m, 1H), 1.01 (d, *J* = 6.7 Hz, 3H). Data is in agreement with that reported in the literature.[6]

(*R*)-4-Benzyl-3-((*R*)-3,7-dimethyloct-6-enoyl)oxazolidin-2-one (21)

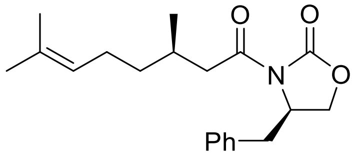

Oxazolidinone **21** was prepared according to General Procedure C from **12** (1.0 g, 5.874 mmol), PvCl (1.06 g, 8.81 mmol), Et_3N (4.1 mL, 29.37 mmol), DMAP (359.2 mg, 2.94 mmol) and **(*R*)-19** (1.04 g, 5.874 mmol) and purified over silica gel (hexane : ethyl acetate = 50/1 to 40/1 to 4/1 and TLC hexane : ethyl acetate = 4 : 1) to give the product as a yellow oil (1.3 g, 68%). ^{1}H NMR (400 MHz, Chloroform-*d*) δ 7.41 – 7.13 (m, 5H), 5.11 (tdq, *J* = 7.0, 2.8, 1.4 Hz, 1H), 4.68 (ddt, *J* = 9.7, 7.2, 3.3 Hz, 1H), 4.23 – 4.05 (m, 2H), 3.36 – 2.91 (m, 2H), 2.80 – 2.69 (m, 2H), 2.17 – 1.93 (m, 1H), 1.69 (d, *J* = 1.4 Hz, 3H), 1.61 (d, *J* = 1.3 Hz, 3H), 1.44 (dddd, *J* = 13.3, 9.5, 6.5, 5.6 Hz, 1H), 1.35 – 1.25 (m, 1H), 0.99 (d, *J* = 6.6 Hz, 3H). Data is in agreement with that reported in the literature.[6]

Synthesis of methylated oxazolidinone (General procedure D)

Sodium hexamethyldisilamide (NaHMDS, 3.6 eq.) was added to a flask containing a solution of **20** or **21** (1 eq.) in dry THF (60 mL) at -78 °C. After 1 h, iodomethane (MeI, 14 eq.) was added dropwise and the solution was stirred for 6 h at -78 °C. The reaction mixture was quenched with glacial acetic acid (AcOH, 2.5 mL) and aqueous NH_4Cl solution (25 ml) at -78 °C and then brought to 0 °C. The aqueous layer was extracted with ethyl acetate (3 x 40 mL). The organic layers were combined and washed with aqueous $Na_2S_2O_3$ solution (50 ml) and brine (50 mL), dried over anhydrous Na_2SO_4, filtered and concentrated under reduced pressure. It was purified over silica gel to give the product.

(*S*)-4-Benzyl-3-((2*S*,3*R*)-2,3,7-trimethyloct-6-enoyl)oxazolidin-2-one (22)

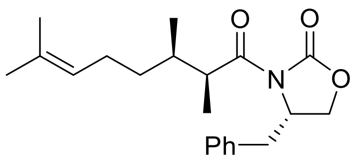

Oxazolidinone **22** was prepared according to General Procedure D from **20** (1.15 g, 3.49 mmol), NaHMDS (13 mL, 12.57 mmol) and MeI (6.9 g, 48.87 mmol) and purified over silica gel (hexane : ethyl acetate = 50 : 1 to 45 : 1 to 30 : 1 to 15 : 1 to 8 : 1 and TLC hexane : ethyl acetate = 6 : 1) to give the product as a yellow oil (880.5 mg, 74%). ^{1}H NMR (300 MHz, Chloroform-*d*) δ 7.40 – 7.15 (m, 5H), 5.09 (dddd, *J* = 8.5, 5.6, 2.9, 1.5 Hz, 1H), 4.65 (ddt, *J* = 9.8, 6.5, 3.3 Hz, 1H), 4.25 – 4.07 (m, 2H), 3.30 (dd, *J* = 13.3, 3.2 Hz, 1H), 2.76 (dd, *J* = 13.3, 9.7 Hz, 1H), 2.13 – 1.76 (m, 3H), 1.67 (d, *J* = 1.3 Hz, 3H), 1.59 (d, *J* = 1.3 Hz, 3H), 1.39 (dddd, *J* = 13.1, 10.7, 6.4, 4.5 Hz, 1H), 1.30 – 1.17 (m, 1H), 1.14 (d, *J* = 6.9 Hz, 3H), 0.90 (d, *J* = 6.8 Hz, 3H). Data is in agreement with that reported in the literature.[6]

(*R*)-4-Benzyl-3-((2*R*,3*R*)-2,3,7-trimethyloct-6-enoyl)oxazolidin-2-one (23)

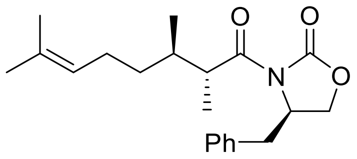

Oxazolidinone **23** was prepared according to General Procedure D from **21** (1.2 g, 3.643 mmol), NaHMDS (13.3 mL, 13.114 mmol) and MeI (7.2 g, 51.0 mmol) and purified over silica gel (hexane : ethyl acetate = 50 : 1 to 45 : 1 to 30 : 1 to 15 : 1 to 8 : 1 and TLC hexane : ethyl acetate = 6 : 1) to give the product as a yellow oil (805.9 mg, 75%). ^{1}H NMR (400 MHz, Chloroform-*d*) δ 7.39 – 7.15 (m, 5H), 5.07 (ddp, *J* = 6.9, 5.7, 1.4 Hz, 1H), 4.72 – 4.61 (m, 1H), 4.22 – 4.08 (m, 2H), 3.29 (dd, *J* = 13.3, 3.3 Hz, 1H), 2.76 (dd, *J* = 13.3, 9.7 Hz, 1H), 2.04 (td, *J* = 12.4, 10.9, 7.3 Hz, 1H), 1.95 – 1.76 (m, 2H), 1.67 (q, *J* = 1.3 Hz, 3H), 1.60 (d, *J* = 1.3 Hz, 3H), 1.48 (dddd, *J* = 13.1, 9.9, 6.8, 3.2 Hz, 1H), 1.17 (d, *J* = 6.9 Hz, 3H), 1.16 – 1.09 (m, 1H), 0.96 (d, *J* = 6.7 Hz, 3H). Data is in agreement with that reported in the literature.[6]

Synthesis of methylated (*R*)-citronellol (General procedure E)

$NaBH_4$ (3 eq.) was added to a solution of **22** or **23** (1 eq.) in dry THF (20 mL) at 0 ºC and then water (8 mL) was added. The solution was warmed to rt and stirred for 6 h. The reaction mixture was quenched with aqueous HCl (1 M 35 mL) and the aqueous layer was extracted with ethyl acetate (3 x 30 mL). The organics were washed with brine (80 ml), dried over anhydrous Na_2SO_4, filtered and concentrated under reduced pressure. The product was purified over silica gel.

(2*S*,3*R*)-2,3,7-Trimethyloct-6-en-1-ol (7)

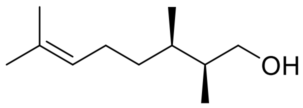

Alcohol **7** was prepared according to General Procedure E from **22** (871 mg, 2.536 mmol), and $NaBH_4$ (287.8 mg, 7.608 mmol) and purified over silica gel (hexane : ethyl acetate = 50 : 1 to 45 : 1 to 30 : 1 to 10 : 1 and TLC hexane : ethyl acetate = 5 : 1) to give the product as a light-yellow oil (302.5 mg, 75%). ^{1}H NMR (300 MHz, Chloroform-*d*) δ 5.10 (tp, *J* = 7.2, 1.6 Hz, 1H), 3.62 – 3.38 (m, 2H), 1.97 (hept, *J* = 7.7 Hz, 2H), 1.68 (d, *J* = 1.5 Hz, 3H), 1.67 – 1.63 (m, 2H), 1.60 (s, 3H), 1.34 (d, *J* = 3.5 Hz, 2H), 1.31 – 1.09 (m, 1H), 0.80 (t, *J* = 6.4 Hz, 6H). Data is in agreement with that reported in the literature.[6]

(2*R*,3*R*)-2,3,7-Trimethyloct-6-en-1-ol (8)

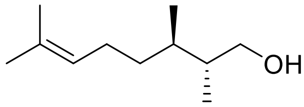

Alcohol **8** was prepared according to General Procedure E from **23** (805.9 mg, 346 mmol), and $NaBH_4$ (287.8 mg, 7.608 mmol) and was purified over silica gel (hexane : ethyl acetate = 50 : 1 to 45 : 1 to 30 : 1 to 10 : 1 and TLC hexane : ethyl acetate = 5 : 1) to give the product as a light-yellow oil (295.0 mg, 74%). ^{1}H NMR (300 MHz, Chloroform-*d*) δ 5.10 (ddq, *J* = 8.5, 5.6, 1.4 Hz, 1H), 3.68 – 3.37 (m, 2H), 1.96 (ddq, *J* = 46.1, 14.8, 7.5 Hz, 2H), 1.68 (q, *J* = 1.3 Hz, 3H), 1.60 (d, *J* = 1.3 Hz, 3H), 1.59 – 1.45

(m, 2H), 1.44 – 1.36 (m, 1H), 1.34 (d, J = 3.5 Hz, 1H), 1.10 (dtd, J = 13.2, 9.4, 5.4 Hz, 1H), 0.89 (dd, J = 6.7, 2.3 Hz, 6H). Data is in agreement with that reported in the literature.[6]

Synthesis of (*R*)-ECO and its derivatives (General procedure F)

Ethyl chloroglyoxylate (2 eq.) was add to a 50 mL flask of (*R*)-citronellol **1** or its derivatives (1 eq.) in Et_3N (3 eq) and DCM (25 mL) solution under N_2. The mixture will be stirred at rt for 3 h and the reaction was quenched with saturated $NaHCO_3$ (15 mL) and washed with water (2 x 20 mL). The aqueous layer was extracted with DCM (3 x 15 mL) and the combine the organic phase were dried over anhydrous Na_2SO_4, concentrated under reduced pressure, and the product was purified over silica gel.

(*R*)-3,7-Dimethyloct-6-en-1-yl ethyl oxalate ((*R*)-ECO, 2)

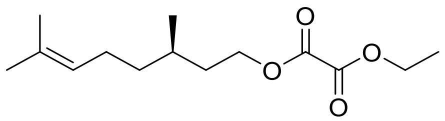

Oxalate ester **2** was prepared according to General Procedure F from **1** (1 g, 6.4 mmol), ethyl chloroglyoxylate (1.75 g, 12.8 mmol) and Et_3N (2.7 mL, 19.2 mmol) and purified over silica gel (hexane : ethyl acetate = 30 : 1 to 25 : 1 to 20 : 1 and TLC hexane : ethyl acetate = 8 : 1) to give the product as a light-yellow oil (0.9 g, 55%). ^1H NMR (500 MHz, Chloroform-*d*) δ 5.07 (dddt, J = 8.5, 7.0, 2.8, 1.4 Hz, 1H), 4.39 – 4.32 (m, 2H), 4.36 – 4.27 (m, 2H), 2.06 – 1.89 (m, 2H), 1.84 – 1.71 (m, 1H), 1.67 (q, J = 1.3 Hz, 3H), 1.59 (d, J = 1.4 Hz, 3H), 1.58 – 1.50 (m, 2H), 1.37 (t, J = 7.1 Hz, 3H), 1.37 – 1.29 (m, 1H), 1.28 – 1.13 (m, 1H), 0.93 (d, J = 6.4 Hz, 3H). $[\alpha]^{20}_D$ = +1.46° (c = 0.1, $CHCl_3$). $[\alpha]^{20}_D$ = +1.46° (c = 0.1, $CHCl_3$); Lit.: $[\alpha]^{25}_D$ = +1.57° (c = 1.07, $CHCl_3$).[7]

Ethyl ((2*S*,3*R*)-2-fluoro-3,7-dimethyloct-6-en-1-yl) oxalate (5)

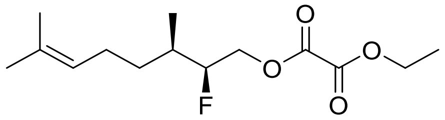

Oxalate ester **5** was prepared according to General Procedure F from **3** (200 mg, 1.14 mmol), ethyl chloroglyoxylate (311.3 mg, 2.28 mmol) and Et_3N (0.48 mL, 3.42 mmol) and purified over silica gel (hexane : ethyl acetate = 25 : 1 to 20 : 1 and TLC hexane : ethyl acetate = 4 : 1) to give the product as a light-yellow oil (148.6 mg, 48%). ^{1}H NMR (400 MHz, Chloroform-*d*) δ 5.07 (tdt, *J* = 7.1, 2.9, 1.4 Hz, 1H), 4.64 (dddd, *J* = 49.2, 7.3, 4.7, 2.4 Hz, 1H), 4.53 – 4.29 (m, 4H), 2.03 (dp, *J* = 22.0, 7.0 Hz, 2H), 1.87 – 1.71 (m, 1H), 1.68 (q, *J* = 1.3 Hz, 3H), 1.60 (d, *J* = 1.4 Hz, 3H), 1.51 (dddd, *J* = 13.5, 9.2, 6.8, 5.2 Hz, 1H), 1.38 (t, *J* = 7.1 Hz, 3H), 1.33 – 1.20 (m, 1H), 0.99 (dd, *J* = 6.8, 1.0 Hz, 3H). ^{13}C NMR (101 MHz, Chloroform-*d*) δ 157.7 (d, *J* = 35.9 Hz), 132.4, 123.8, 94.4, 92.6, 67.1 (d, *J* = 23.5 Hz), 63.5, 34.4 (d, *J* = 19.3 Hz), 32.6 (d, *J* = 3.3 Hz), 25.8, 25.3, 17.8, 14.1, 13.9 (d, *J* = 6.3 Hz). ^{19}F NMR (377 MHz, Chloroform-*d*) δ -195.68. IR (neat): 1155, 1179, 1742, 1769 cm^{-1}. HRMS (ES$^+$) *m/z*: $[M + Na]^+$ Calculated for $C_{14}H_{23}O_4FNa$ 297.1478; Found 297.1463. $[\alpha]^{20}_D$ = +26.50° (c = 0.05, $CHCl_3$)

Ethyl ((2*R*,3*R*)-2-fluoro-3,7-dimethyloct-6-en-1-yl) oxalate (6)

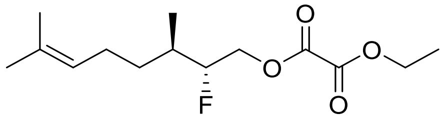

Oxalate ester **6** was prepared according to General Procedure F from **4** (204 mg, 1.17 mmol), ethyl chloroglyoxylate (319.6 mg, 2.34 mmol) and Et_3N (0.49 mL, 3.51 mmol) and purified over silica gel (hexane : ethyl acetate = 25 : 1 to 20 : 1 and TLC hexane : ethyl acetate = 4 : 1) to give the product as a light-yellow oil (160.5 mg, 50%). ^{1}H NMR (500 MHz, Chloroform-*d*) δ 5.08 (tp, *J* = 6.9, 1.5 Hz, 1H), 4.64 – 4.49 (m, 1H), 4.47 – 4.39 (m, 2H), 4.37 (q, *J* = 7.1 Hz, 2H), 2.15 – 1.93 (m, 2H), 1.93 – 1.84 (m, 1H), 1.68 (q, *J* = 1.3 Hz, 3H), 1.60 (d, *J* = 1.4 Hz, 3H), 1.59 – 1.52 (m, 1H), 1.38 (t, *J* = 7.2 Hz, 3H), 1.25 (dtd, *J* = 13.5, 9.2, 5.4 Hz, 1H), 0.97 (d, *J* = 6.9 Hz, 3H). ^{13}C NMR (101 MHz, Chloroform-*d*) δ 157.7 (d, *J* = 38.9 Hz), 132.3, 123.9, 95.1, 93.4, 66.7 (d, *J* = 22.8 Hz), 63.5, 34.4 (d, *J* = 18.9 Hz), 31.7 (d, *J* = 5.5 Hz), 25.8, 25.2, 17.8, 14.9 (d, *J* = 5.3 Hz), 14.1. ^{19}F NMR (470 MHz, Chloroform-*d*) δ -189.59. IR (neat): 1155, 1179, 1742, 1769

cm^{-1}. HRMS (ES$^+$) *m/z*: [M + Na]$^+$ Calculated for $C_{14}H_{23}O_4FNa$ 297.1478; Found 297.1464. $[\alpha]^{20}_D$ = -5.79° (c = 0.1, $CHCl_3$).

Ethyl ((2*S*,3*R*)-2,3,7-trimethyloct-6-en-1-yl) oxalate (9)

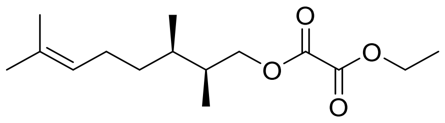

Oxalate ester **9** was prepared according to General Procedure F from **7** (250 mg, 1.468 mmol), ethyl chloroglyoxylate (400.85 mg, 2.936 mmol) and Et_3N (0.62 mL, 4.404 mmol) and purified over silica gel (hexane : ethyl acetate = 50 : 1 to 40 : 1 to 20 : 1 and TLC hexane : ethyl acetate = 5 : 1) to give the product as a light-yellow oil (273.1 mg, 68%). ^{1}H NMR (300 MHz, Chloroform-*d*) δ 5.08 (ddq, *J* = 7.1, 5.7, 1.4 Hz, 1H), 4.35 (qd, *J* = 7.1, 1.1 Hz, 2H), 4.15 (qd, 2H), 2.10 – 1.84 (m, 3H), 1.68 (q, *J* = 1.3 Hz, 3H), 1.60 (d, *J* = 1.3 Hz, 3H), 1.38 (t, *J* = 7.2 Hz, 3H), 1.35 – 1.13 (m, 3H), 0.87 (dd, *J* = 7.0, 1.0 Hz, 3H), 0.83 (dd, *J* = 6.8, 1.1 Hz, 3H). ^{13}C NMR (101 MHz, Chloroform-*d*) δ 158.2, 158.1, 131.7, 124.5, 70.8, 63.2, 36.0, 34.8, 33.4, 25.9 (d, *J* = 9.3 Hz), 17.8, 14.6, 14.1, 11.7. IR (neat): 1155, 1179, 1742, 1769 cm^{-1}. HRMS (ES$^+$) *m/z*: [M + Na]$^+$ Calculated for $C_{15}H_{26}O_4Na$ 293.1729; Found 293.1716. $[\alpha]^{20}_D$ = +11.97° (c = 0.15, $CHCl_3$).

Ethyl ((2*R*,3*R*)-2,3,7-trimethyloct-6-en-1-yl) oxalate (10)

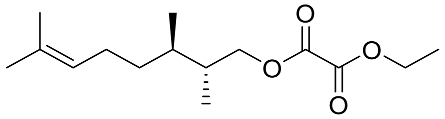

Oxalate ester **10** was prepared according to General Procedure F from **8** (198.7 mg, 1.17 mmol), ethyl chloroglyoxylate (318.6 mg, 2.3 mmol) and Et_3N (0.49 mL, 3.5 mmol) and purified over silica gel (hexane : ethyl acetate = 50 : 1 to 40 : 1 to 20 : 1 and TLC hexane : ethyl acetate = 5 : 1) to give the product as a light-yellow oil (225.3 mg, 71%). ^{1}H NMR (300 MHz, Chloroform-*d*) δ 5.08 (tp, *J* = 7.0, 1.4 Hz, 1H), 4.35 (q, *J* = 7.1 Hz, 2H), 4.31 – 4.03 (m, 2H), 2.11 – 1.97 (m, 1H), 1.90 (dddd, *J* = 10.1, 7.7, 6.2, 4.4 Hz, 2H), 1.68 (q, *J* = 1.3 Hz, 3H), 1.60 (d, *J* = 1.3 Hz, 3H), 1.53 (ddt, *J* = 8.6, 6.9,

3.4 Hz, 1H), 1.45 – 1.41 (m, 1H), 1.37 (t, *J* = 7.1 Hz, 3H), 1.15 (dtd, *J* = 13.3, 9.4, 5.5 Hz, 1H), 0.94 (d, *J* = 6.9 Hz, 3H), 0.91 (d, *J* = 6.9 Hz, 3H). ^{13}C NMR (101 MHz, Chloroform-*d*) δ 158.0 (d, *J* = 23.1 Hz), 131.6, 124.5, 70.0, 63.1, 36.9, 34.4, 33.0, 25.8 (d, *J* = 8.0 Hz), 17.7, 16.4, 13.9 (d, *J* = 2.9 Hz). IR (neat): 1155, 1179, 1742, 1768 cm^{-1}. HRMS (ES^+) *m/z*: $[M + Na]^+$ Calculated for $C_{15}H_{26}O_4Na$ 293.1729; Found 293.1716. $[\alpha]^{20}_D$ = +3.83° (c = 0.15, $CHCl_3$).

NMR Spectra

(*R*)-3,7-Dimethyloct-6-enoic acid ((*R*)-citronellic acid, 12)

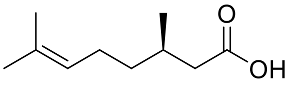

^{1}H NMR (400 MHz, Chloroform-*d*)

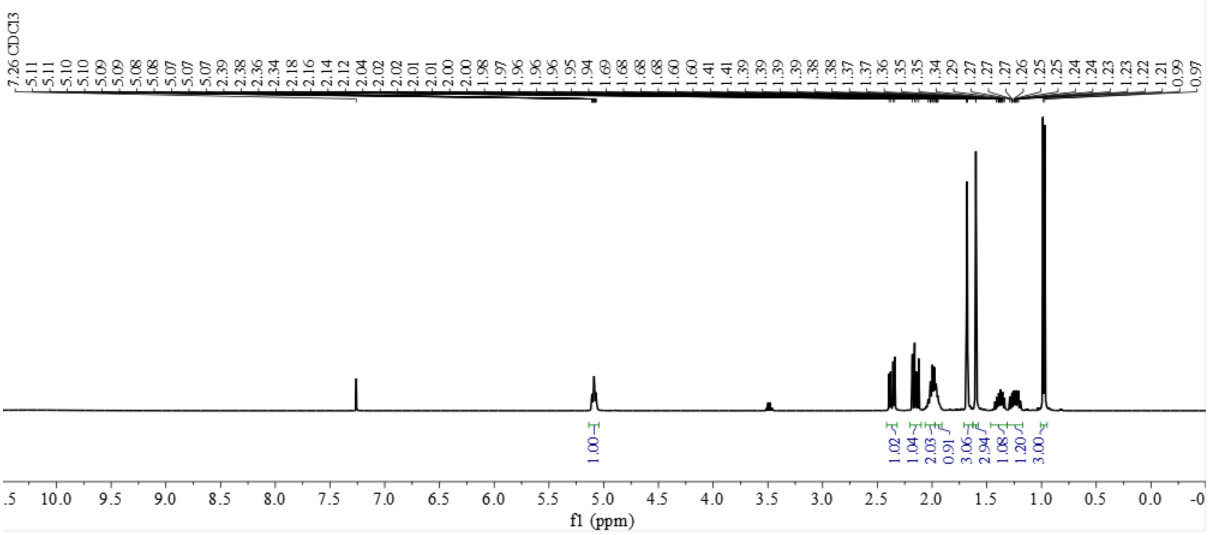

^{13}C NMR (101 MHz, Chloroform-*d*)

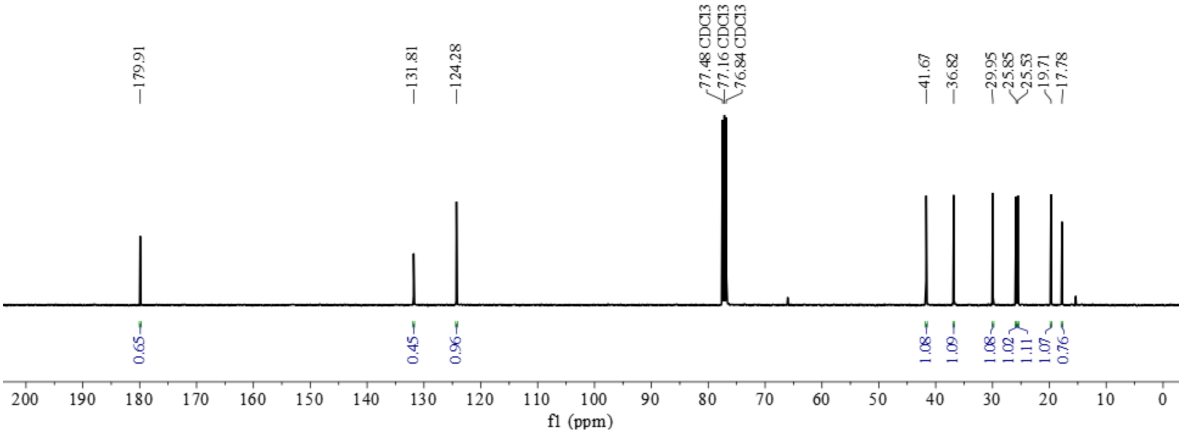

Data is in agreement with that reported in the literature.[2]

(*R*)-3,7-Dimethyloct-6-en-1-ol ((*R*)-citronellol, 1)

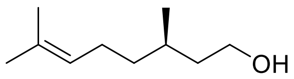

^{1}H NMR (400 MHz, Chloroform-*d*)

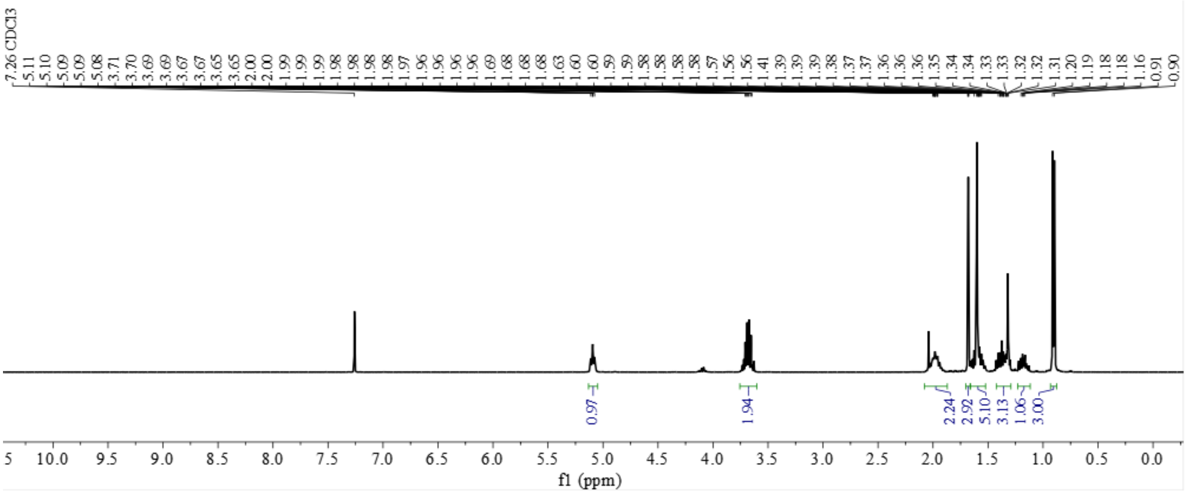

^{13}C NMR (101 MHz, Chloroform-*d*)

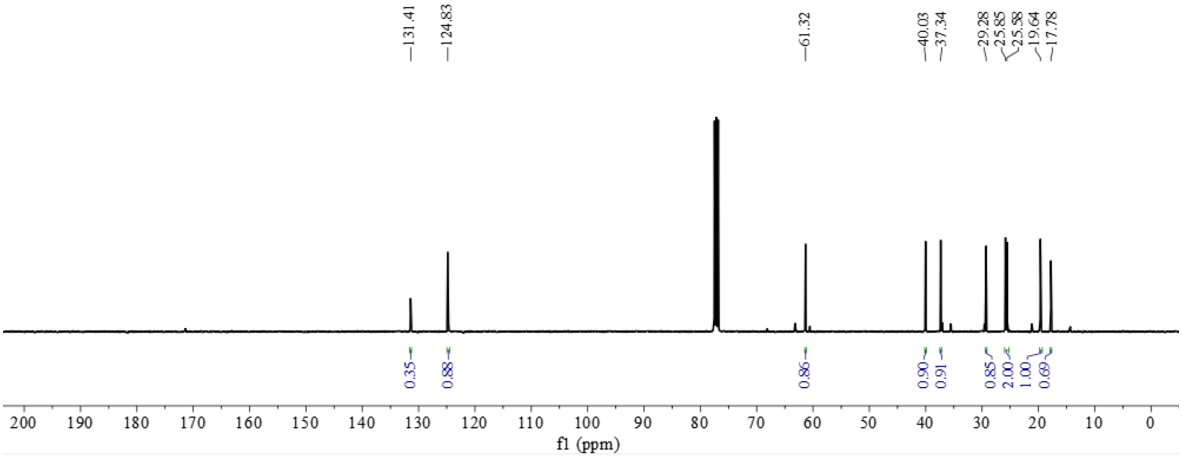

Data is in agreement with that reported in the literature.[3]

(*R*)-3,7-Dimethyloct-6-enal ((*R*)-citronellal, 13)

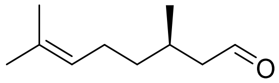

^{1}H NMR (300 MHz, Chloroform-*d*)

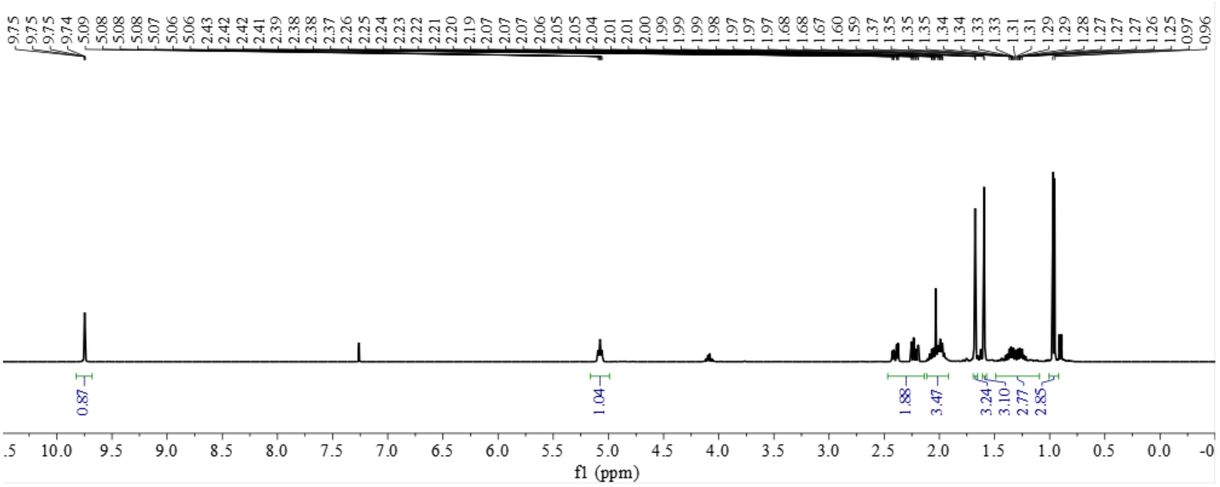

^{13}C NMR (101 MHz, Chloroform-*d*)

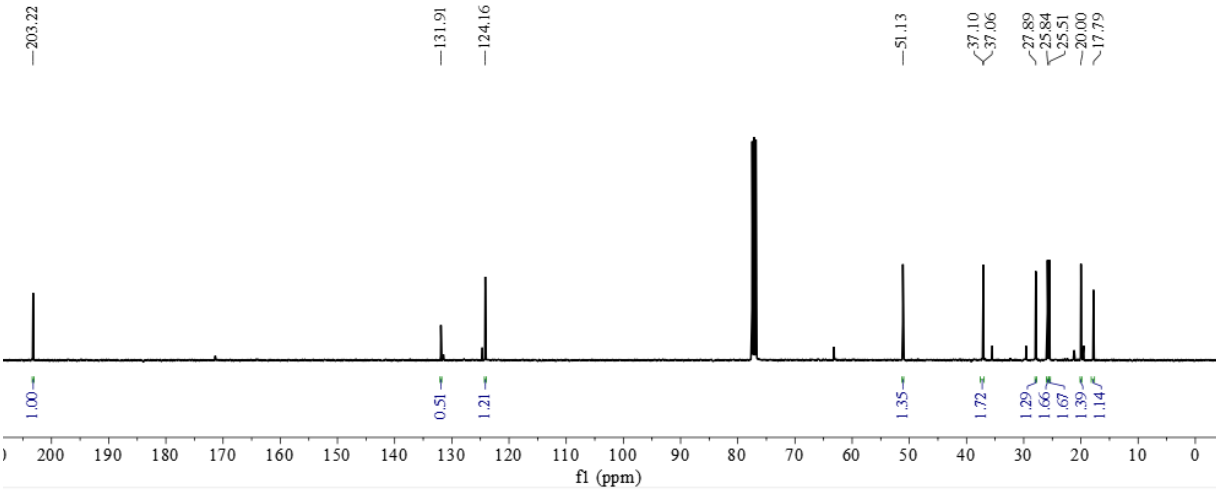

Data is in agreement with that reported in the literature.[3]

(*S*)-5-Benzyl-2,2,3-trimethylimidazolidin-4-one ((*S*)-14c)

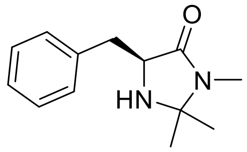

^{1}H NMR (400 MHz, Chloroform-*d*)

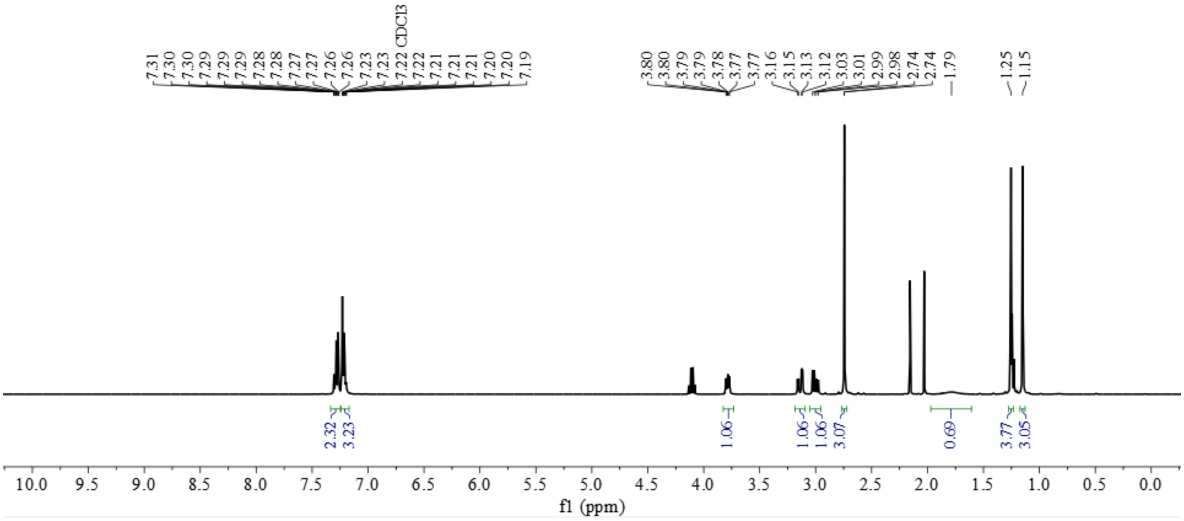

Data is in agreement with that reported in the literature.[4,5]

(*R*)-5-Benzyl-2,2,3-trimethylimidazolidin-4-one (*R*-14c)

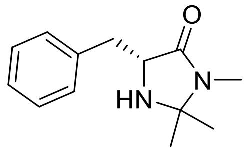

^{1}H NMR (500 MHz, Chloroform-*d*)

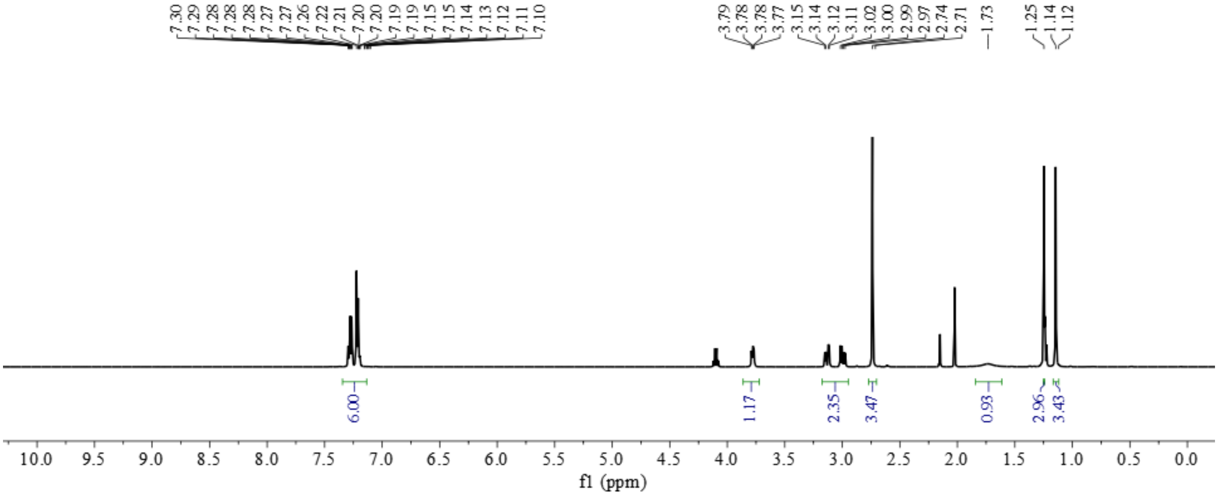

Data is in agreement with that reported in the literature. [4,5]

(2*S*,3*R*)-2-Fluoro-3,7-dimethyloct-6-en-1-ol (3)

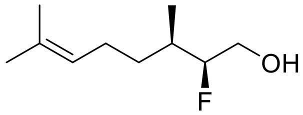

^{1}H NMR (300 MHz, Chloroform-*d*)

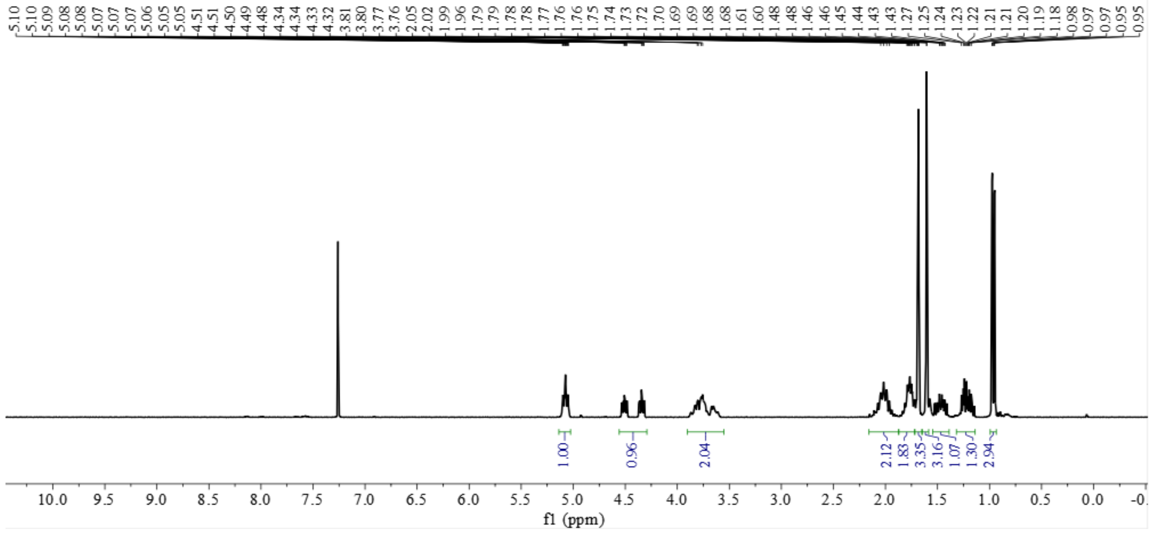

^{13}C NMR (126 MHz, Chloroform-*d*)

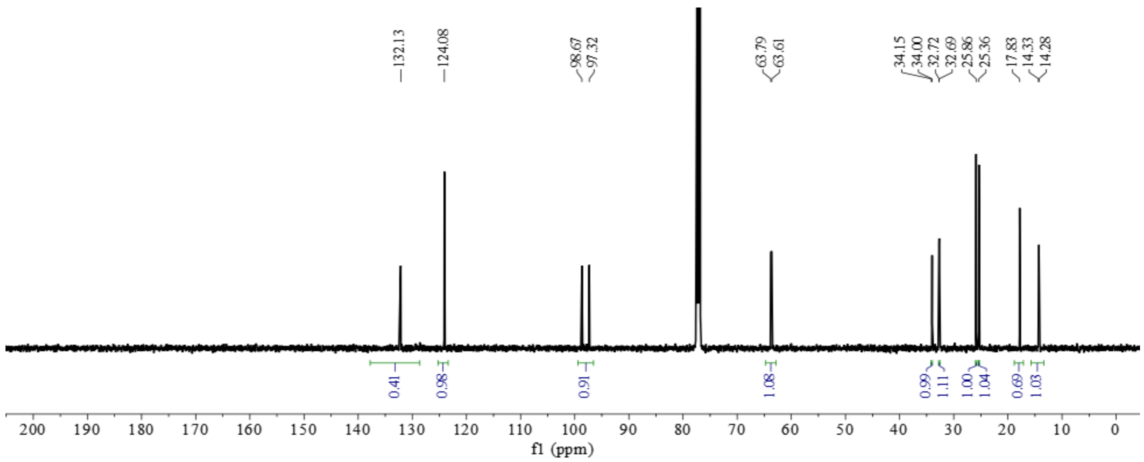

^{19}F NMR (282 MHz, Chloroform-*d*)

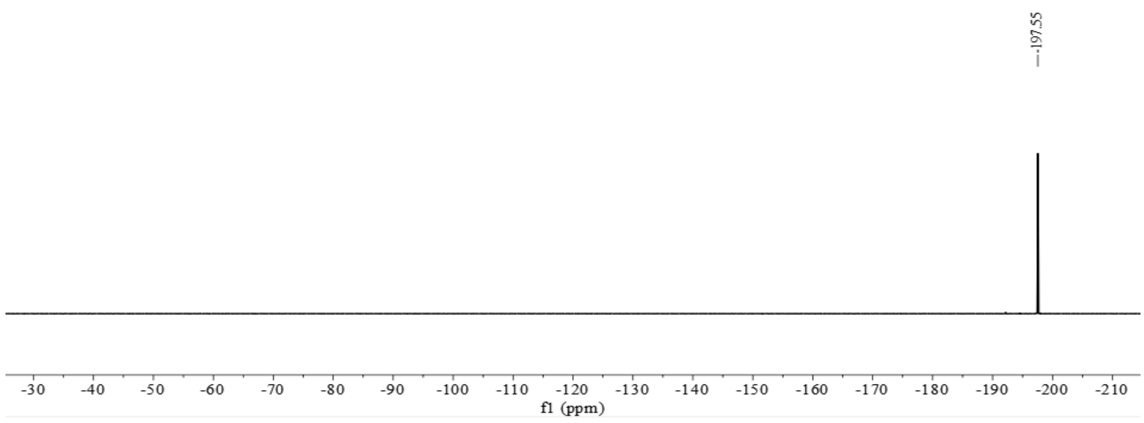

^{1}H-^{1}H-COSY (300 MHz, Chloroform-*d*)

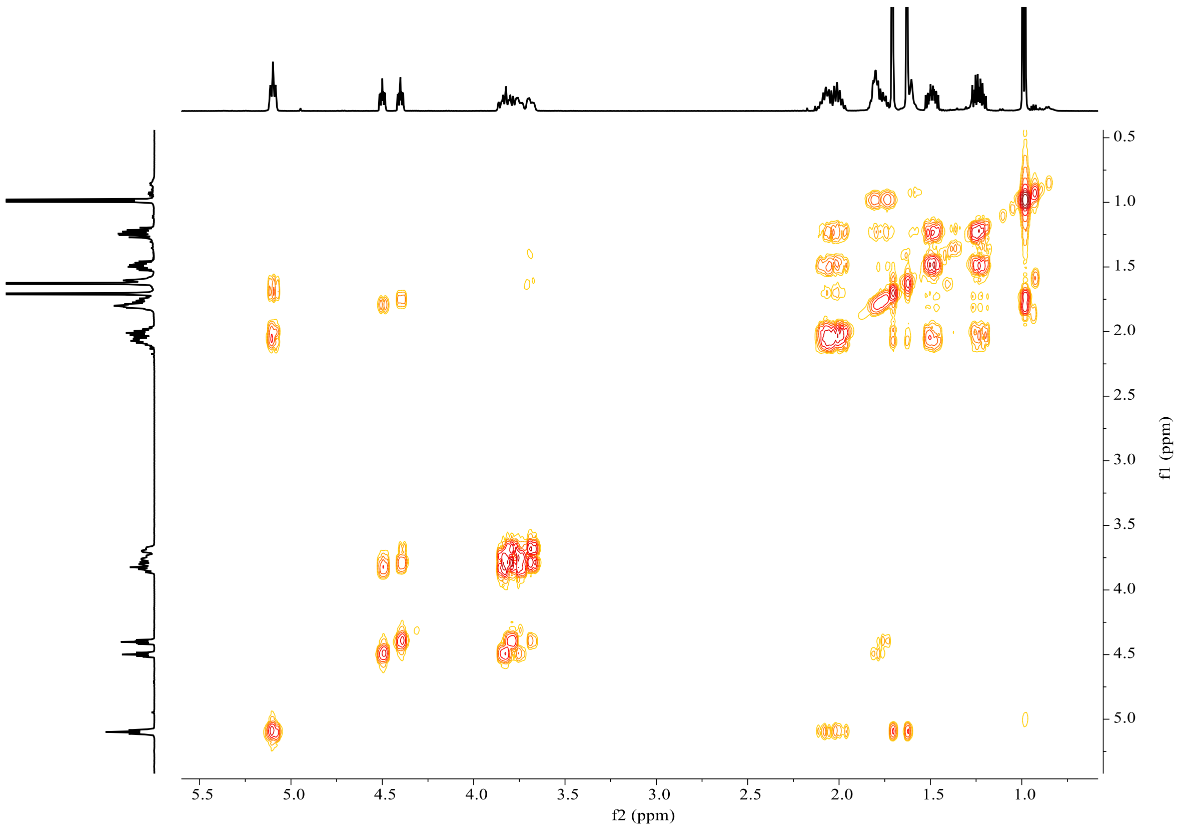

^{1}H-^{13}C-HSQC (300 MHz and 126 MHz, Chloroform-*d*)

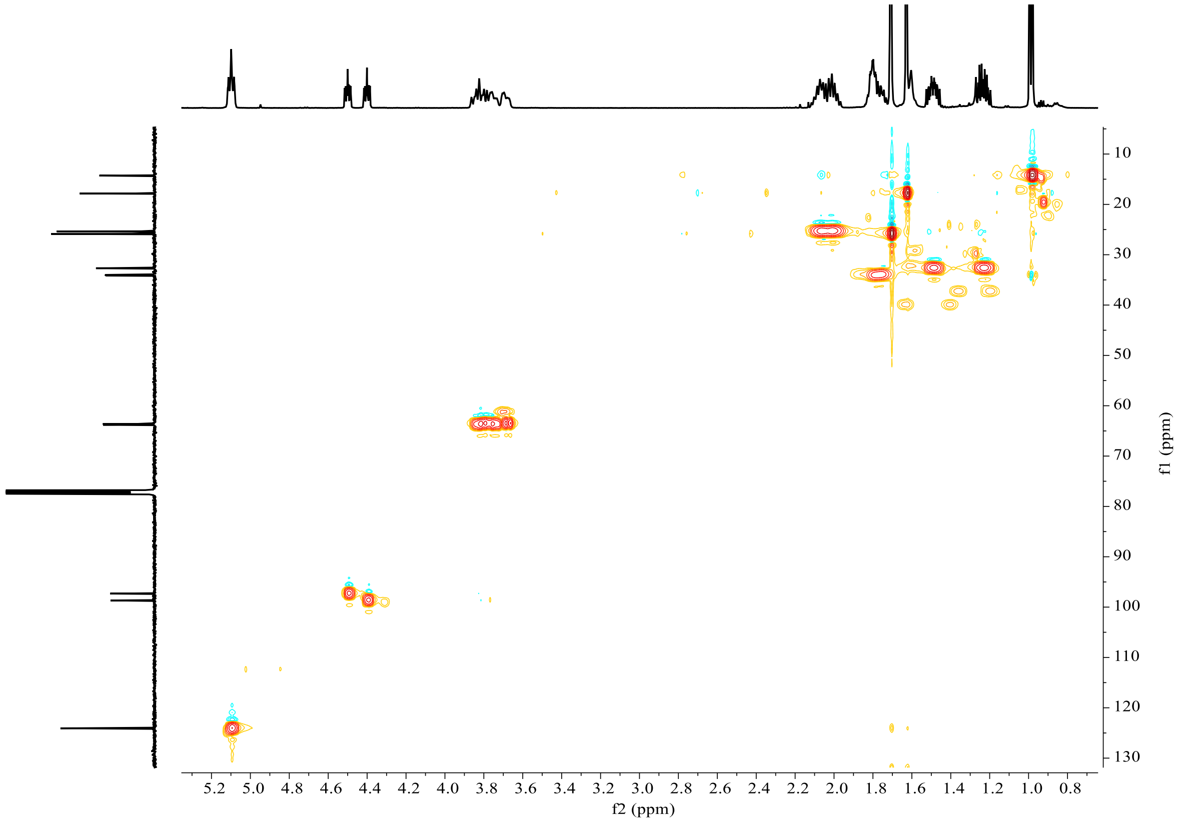

$^{1}H-^{13}C$-HMBC (300 MHz and 126 MHz, Chloroform-*d*)

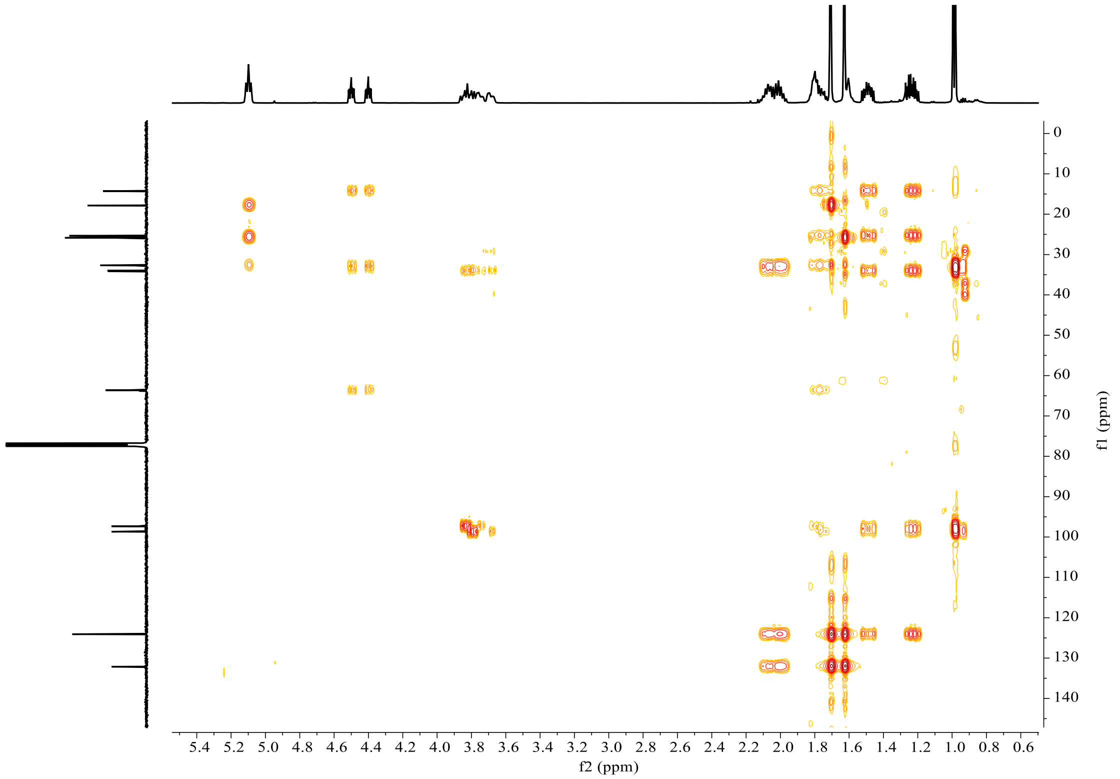

(2*R*,3*R*)-2-Fluoro-3,7-dimethyloct-6-en-1-ol (4)

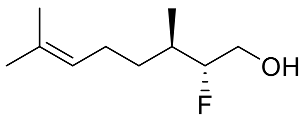

^{1}H NMR (500 MHz, Chloroform-*d*)

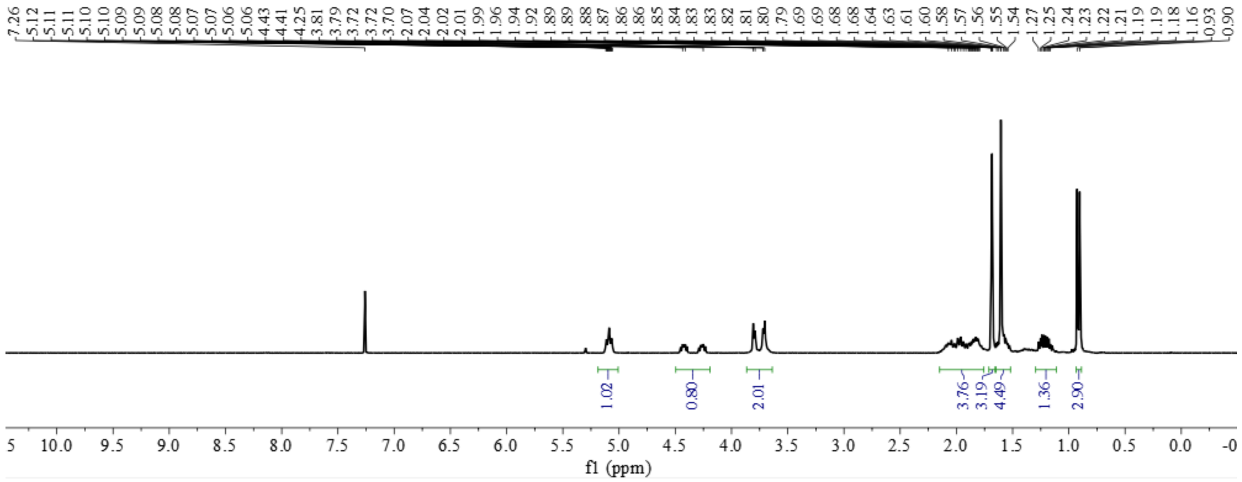

^{13}C NMR (126 MHz, Chloroform-*d*)

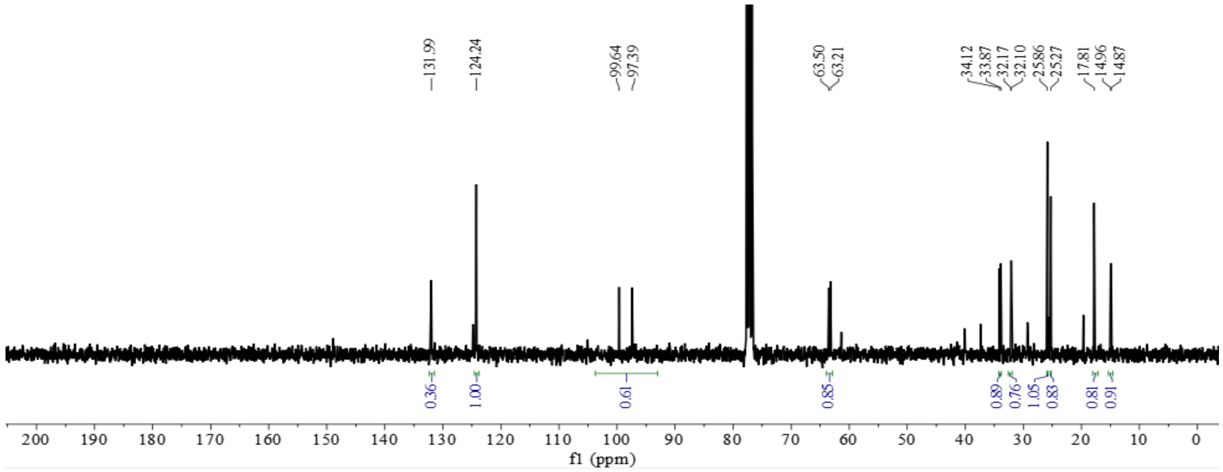

^{19}F NMR (282 MHz, Chloroform-*d*)

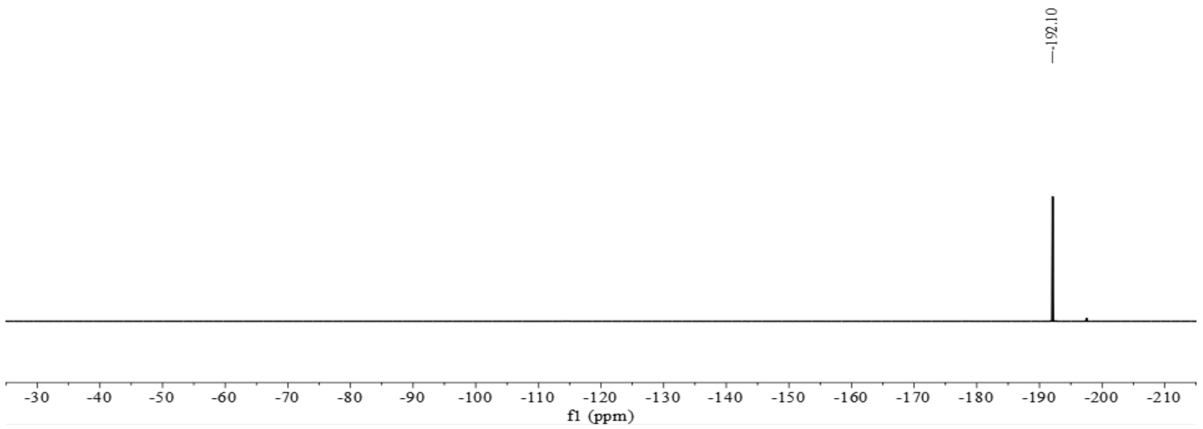

^{1}H-^{1}H-COSY (500 MHz, Chloroform-*d*)

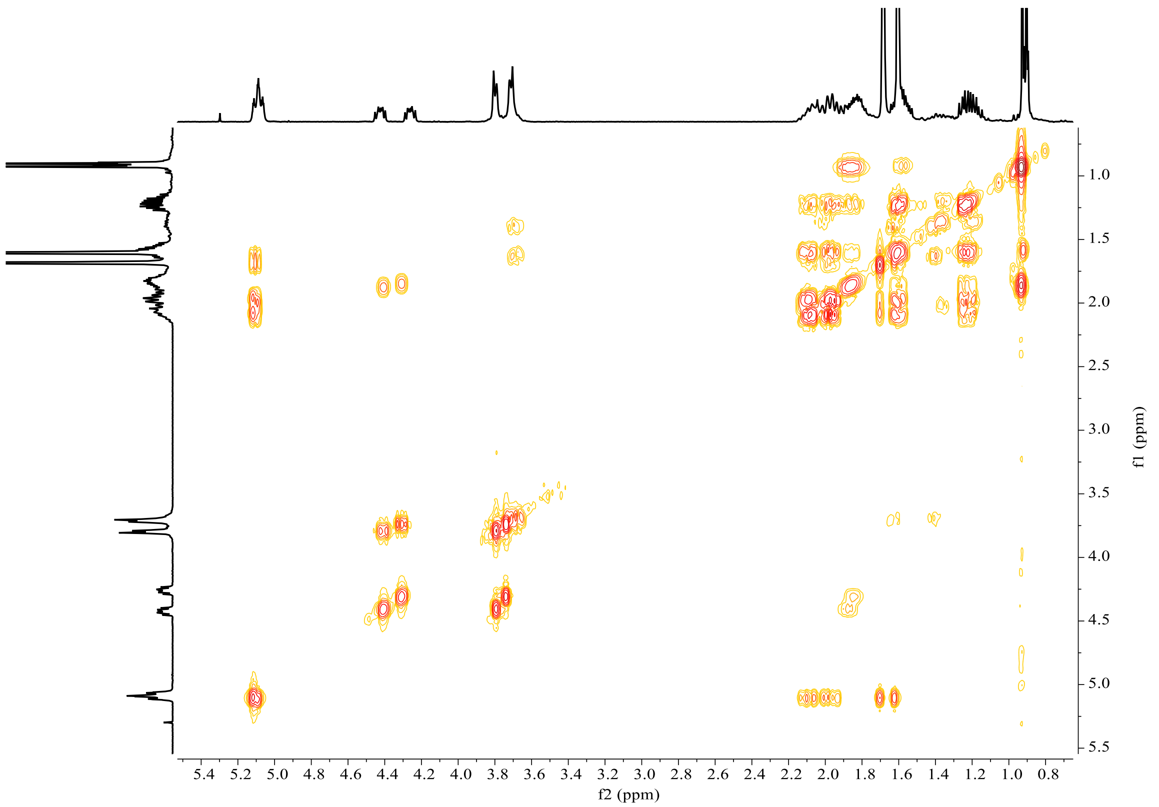

^{1}H-^{13}C-HSQC (500 MHz and 126 MHz, Chloroform-*d*)

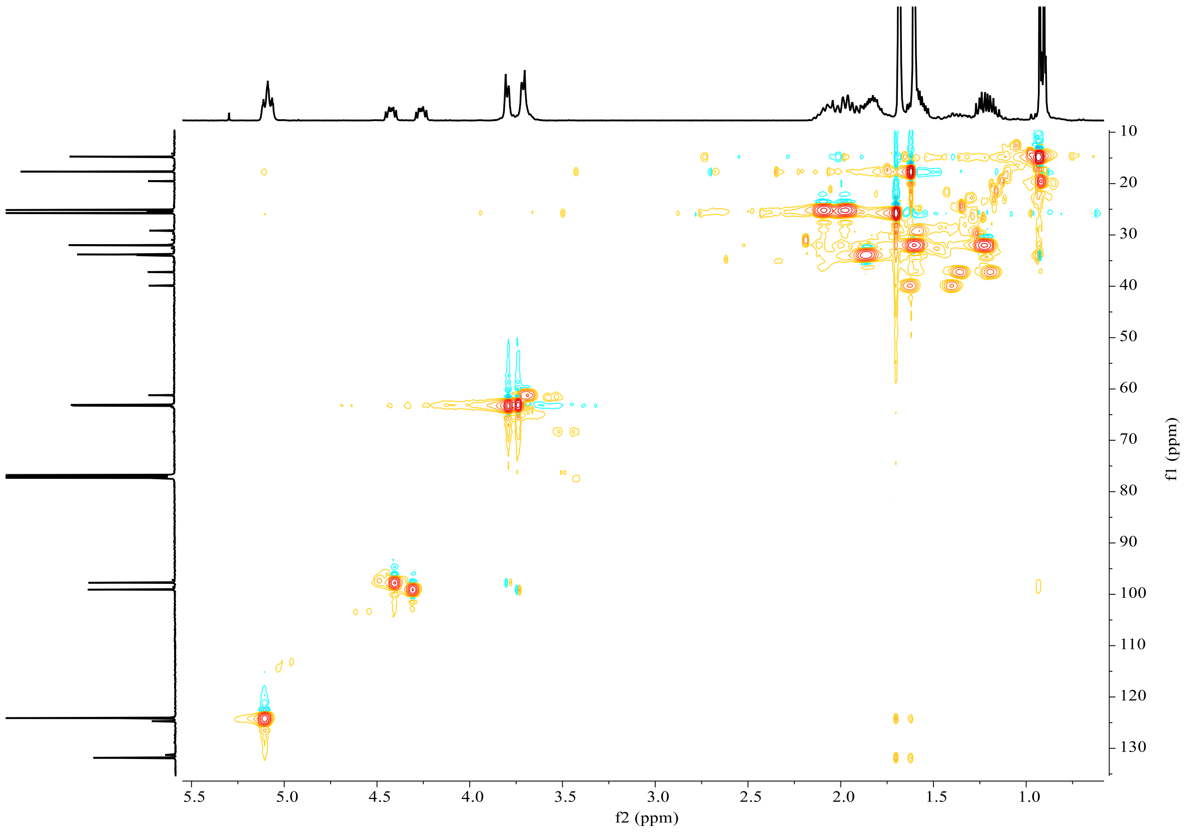

^{1}H-^{13}C-HMBC (500 MHz and 126 MHz, Chloroform-*d*)

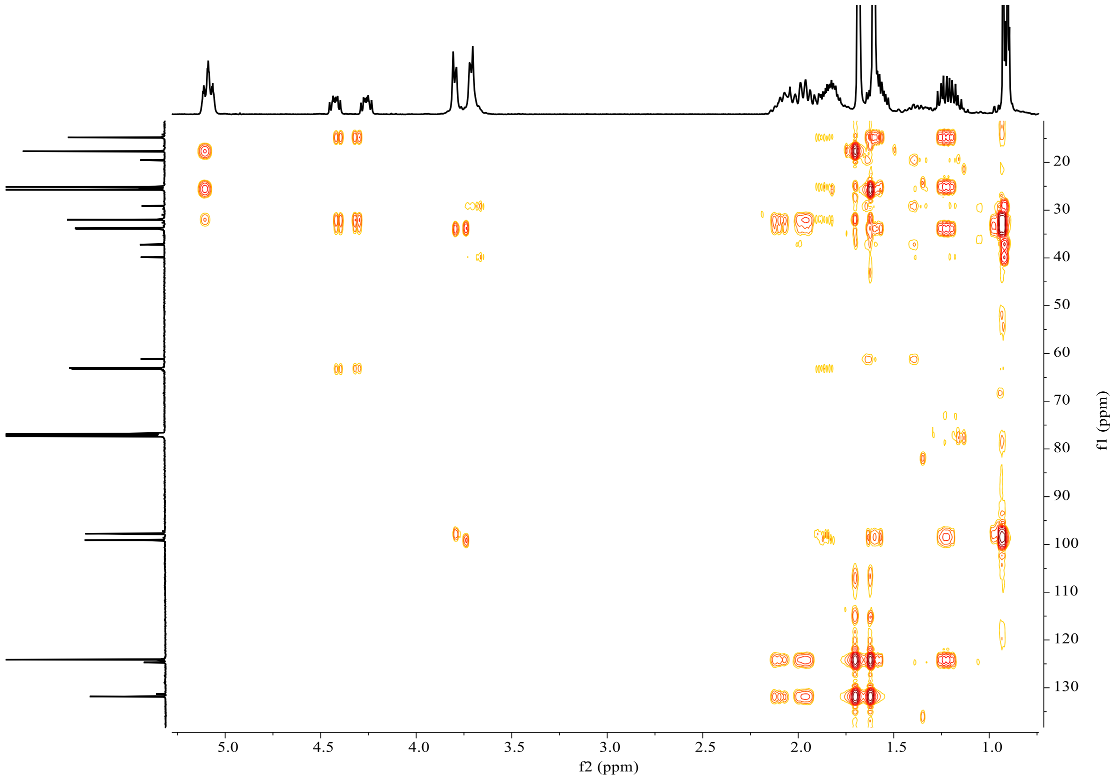

(*R*)-2,2-Difluoro-3,7-dimethyloct-6-en-1-ol (18)

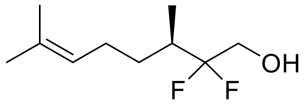

^{1}H NMR (300 MHz, Chloroform-*d*)

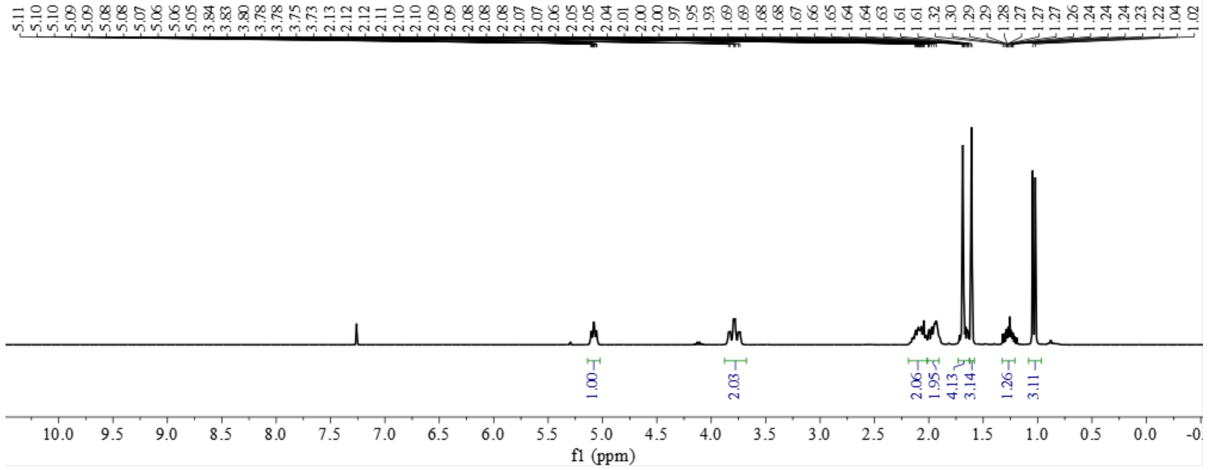

^{13}C NMR (126 MHz, Chloroform-*d*)

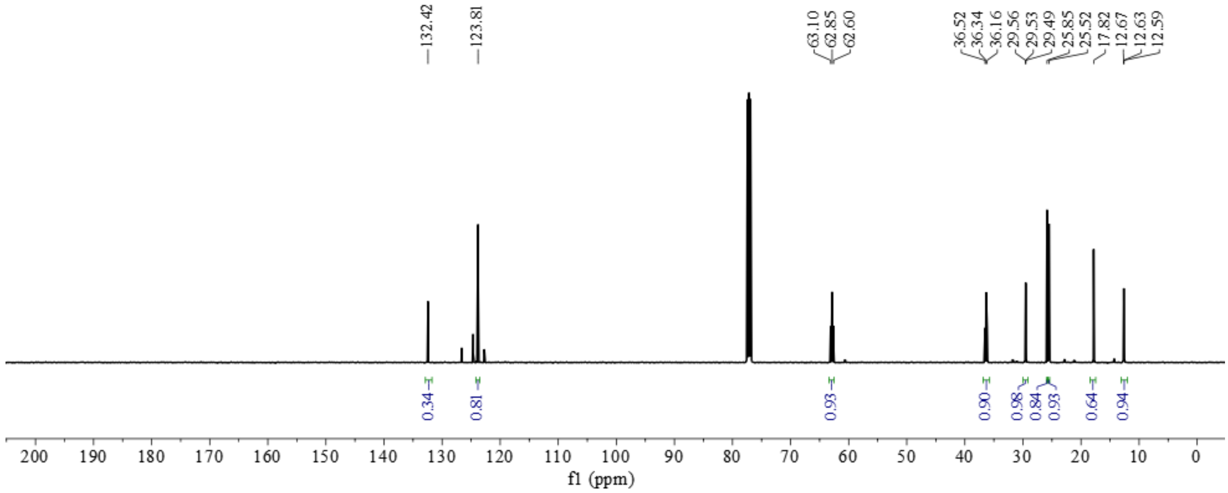

^{19}F NMR (282 MHz, Chloroform-*d*)

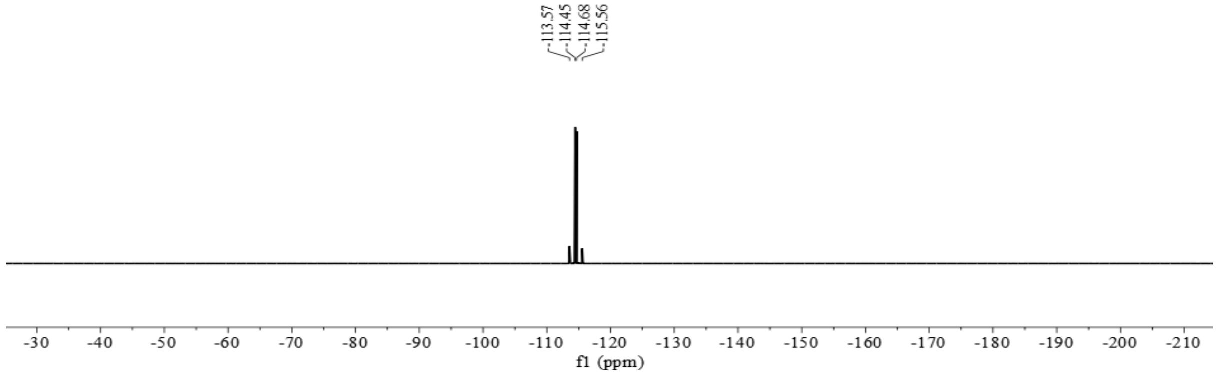

(*S*)-4-Benzyl-3-((*R*)-3,7-dimethyloct-6-enoyl)oxazolidin-2-one (20)

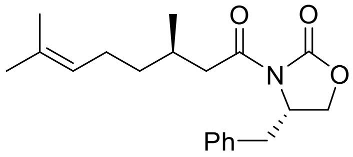

^{1}H NMR (300 MHz, Chloroform-*d*)

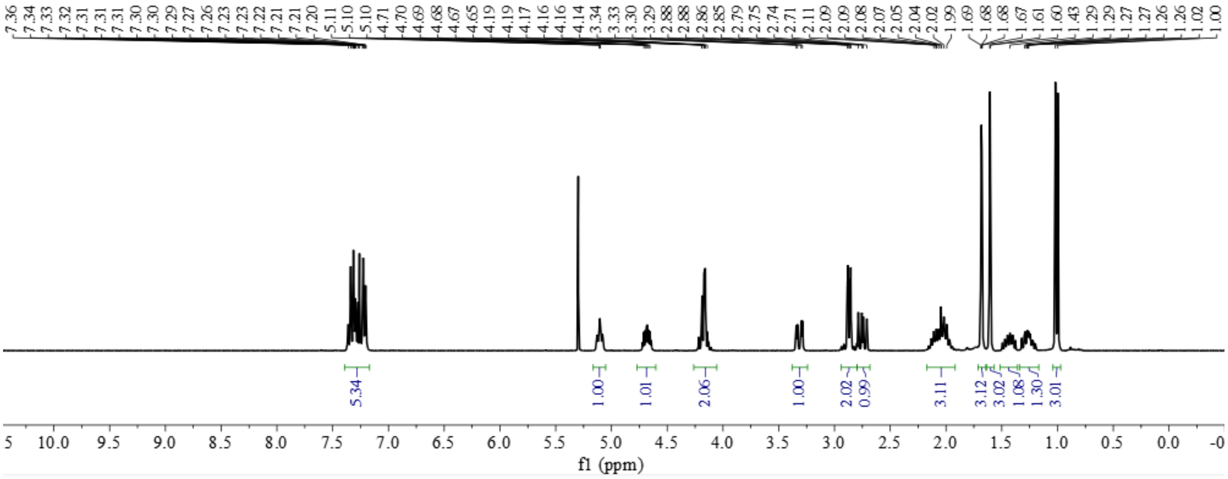

Data is in agreement with that reported in the literature.[6]

(*R*)-4-Benzyl-3-((*R*)-3,7-dimethyloct-6-enoyl)oxazolidin-2-one (21)

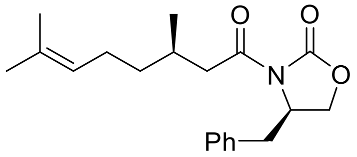

^{1}H NMR (400 MHz, Chloroform-*d*)

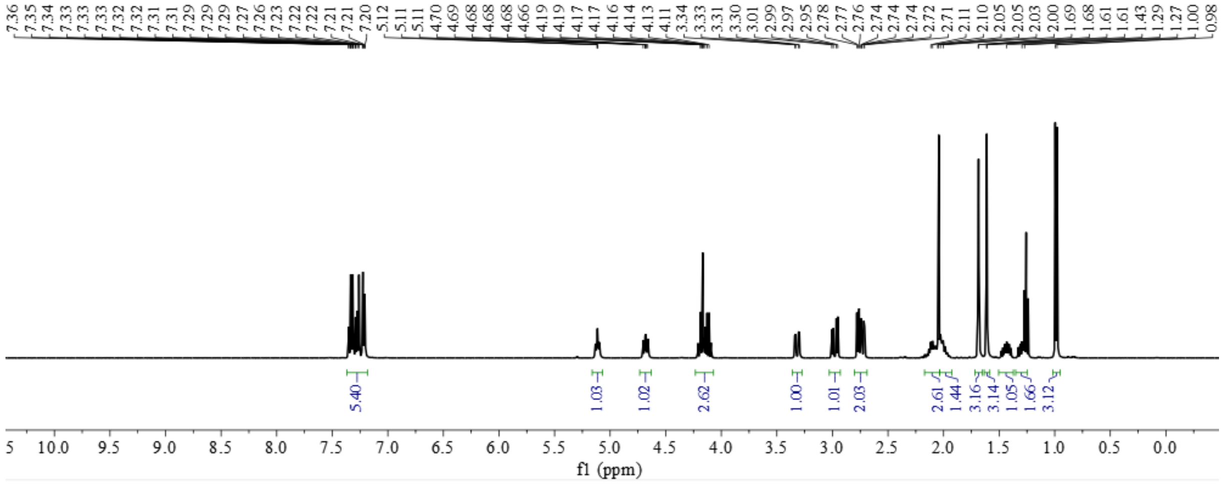

Data is in agreement with that reported in the literature.[6]

(*S*)-4-Benzyl-3-((2*S*,3*R*)-2,3,7-trimethyloct-6-enoyl)oxazolidin-2-one (22)

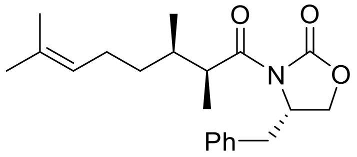

^{1}H NMR (300 MHz, Chloroform-*d*)

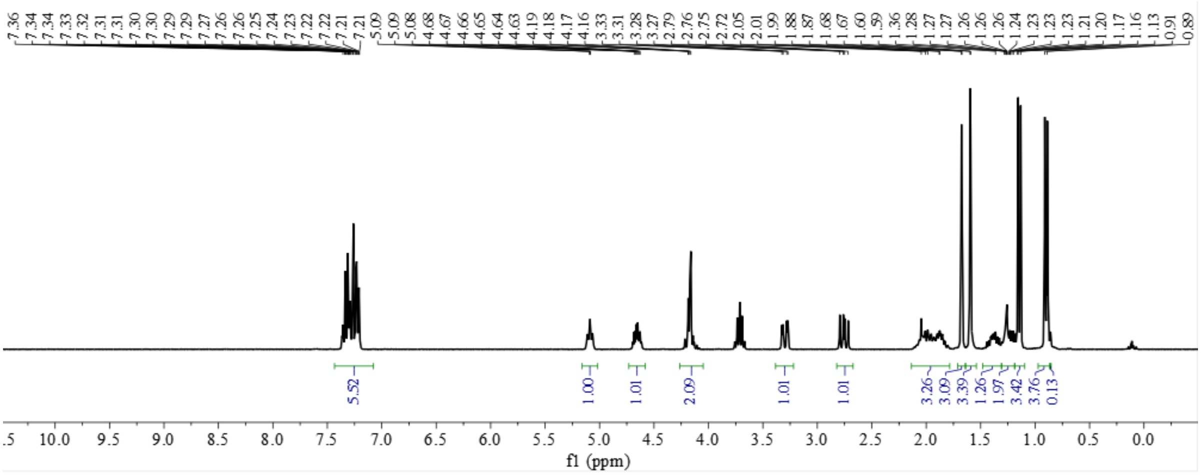

Data is in agreement with that reported in the literature.[6]

(*R*)-4-Benzyl-3-((2*R*,3*R*)-2,3,7-trimethyloct-6-enoyl)oxazolidin-2-one (23)

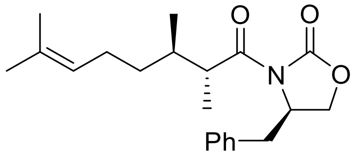

^{1}H NMR (400 MHz, Chloroform-*d*)

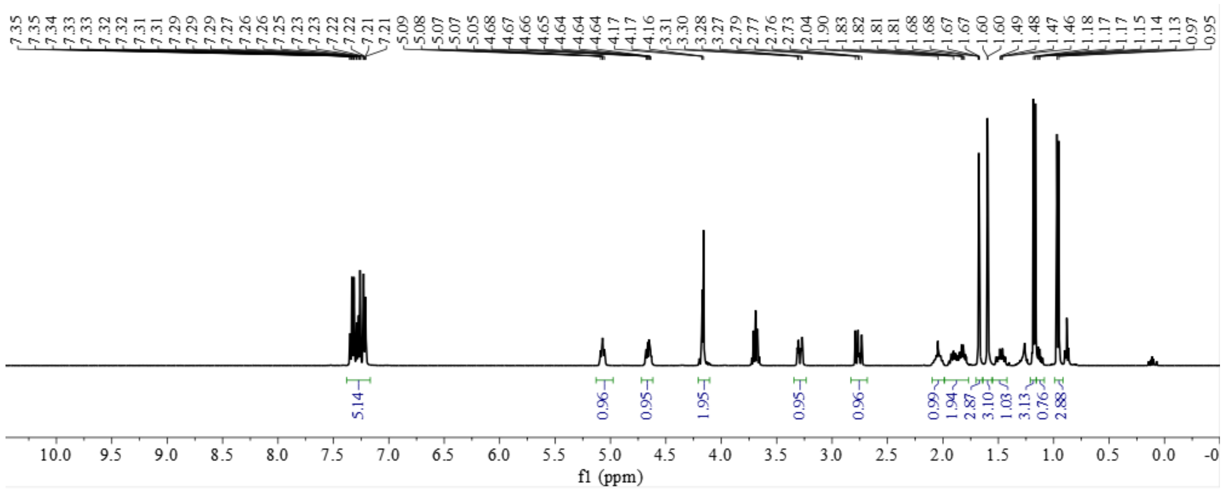

Data is in agreement with that reported in the literature.[6]

(2*S*,3*R*)-2,3,7-Trimethyloct-6-en-1-ol (7)

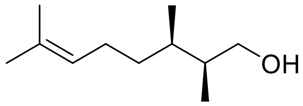

^{1}H NMR (300 MHz, Chloroform-*d*)

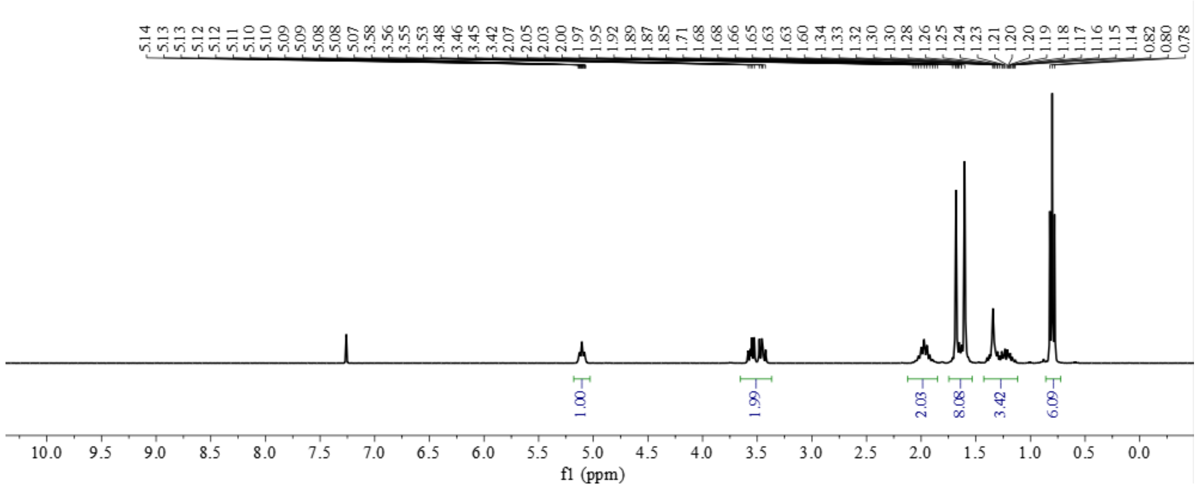

Data is in agreement with that reported in the literature.[6]

(2*R*,3*R*)-2,3,7-Trimethyloct-6-en-1-ol (8)

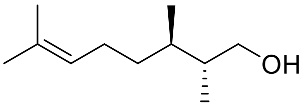

^{1}H NMR (300 MHz, Chloroform-*d*)

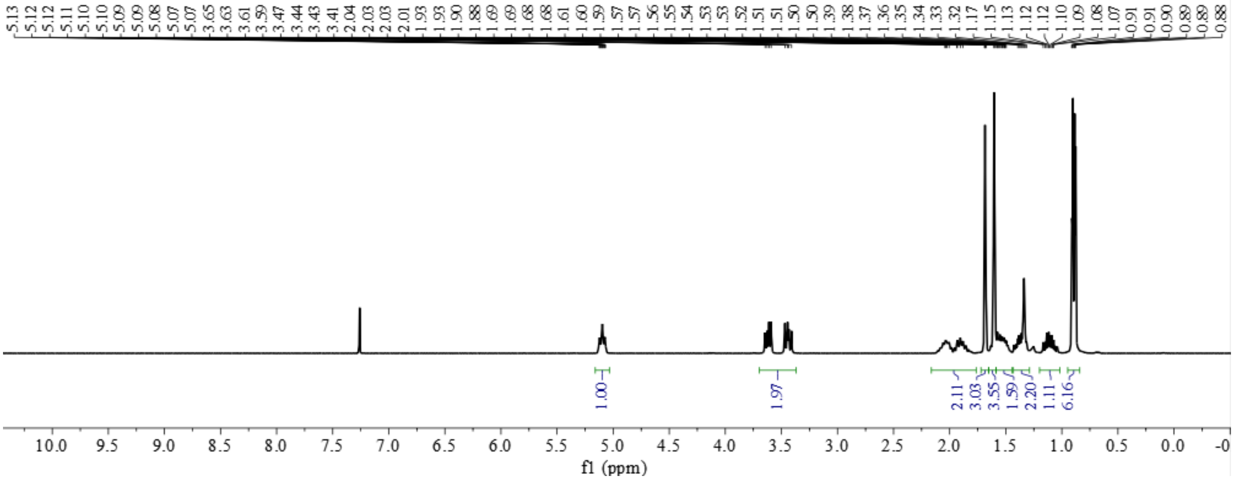

Data is in agreement with that reported in the literature.[6]

(*R*)-3,7-Dimethyloct-6-en-1-yl ethyl oxalate ((*R*)-CEO, 2)

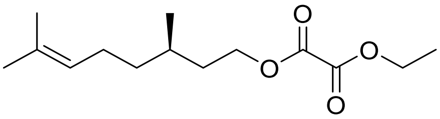

^{1}H NMR (500 MHz, Chloroform-*d*)

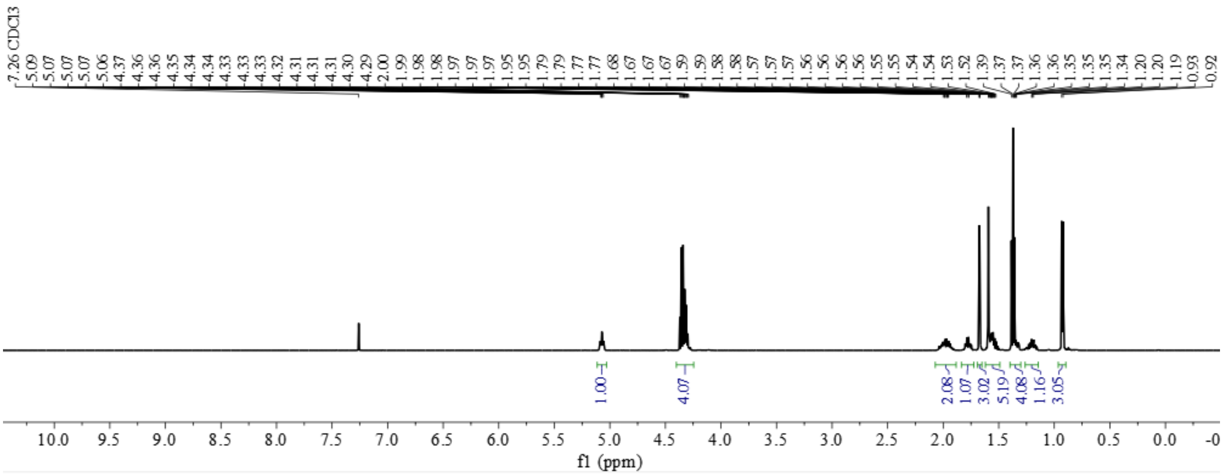

^{13}C NMR (101 MHz, Chloroform-*d*)

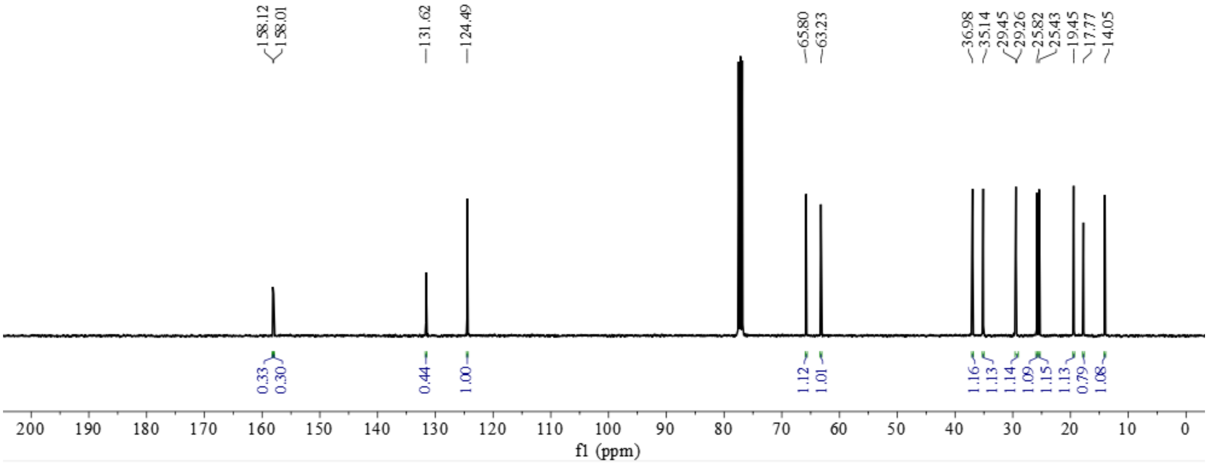

^{13}C DEPTQ (126 MHz, Chloroform-*d*)

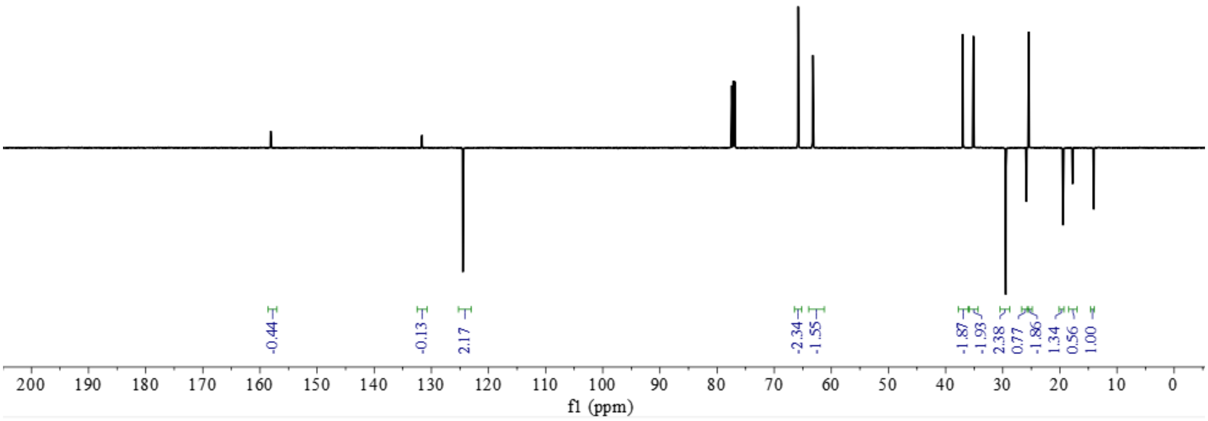

^{1}H-^{13}C-HSQC (500 MHz, 126 MHz, Chloroform-*d*)

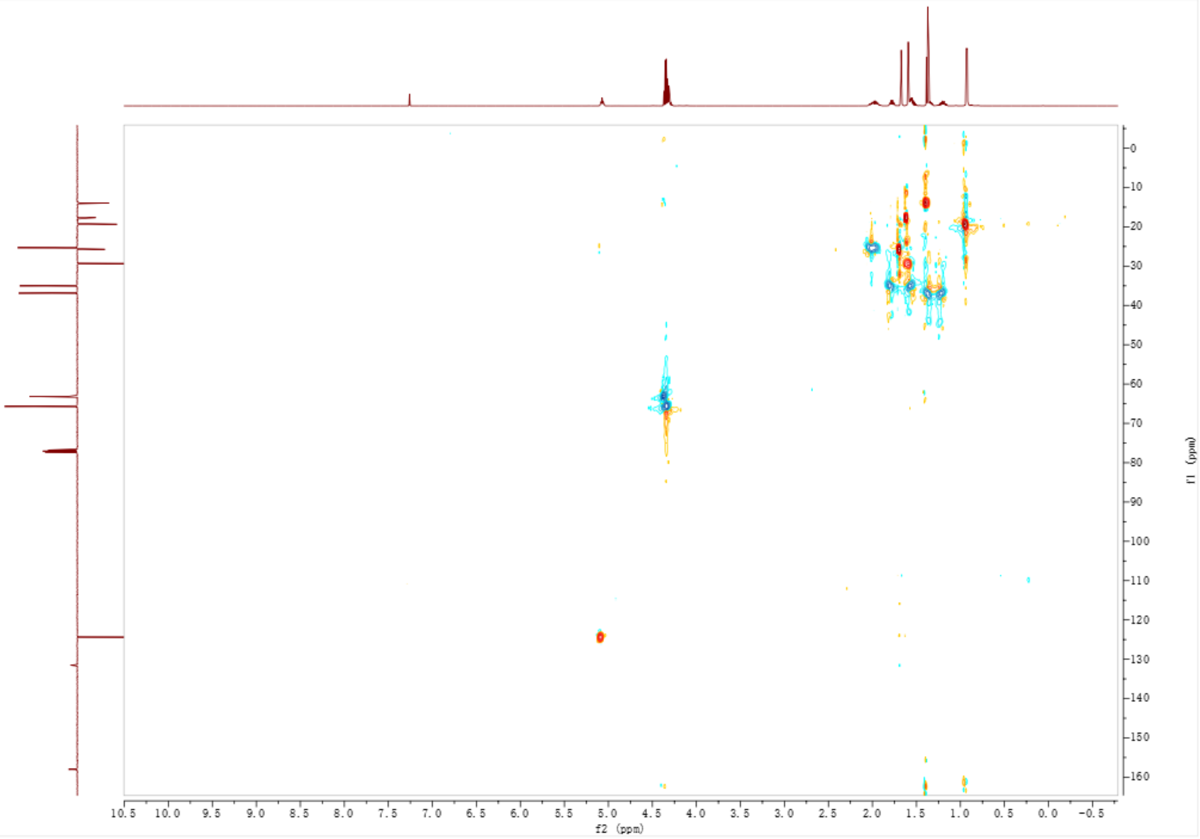

Data is in agreement with that reported in the literature.[7]

Ethyl ((2*S*,3*R*)-2-fluoro-3,7-dimethyloct-6-en-1-yl) oxalate (5)

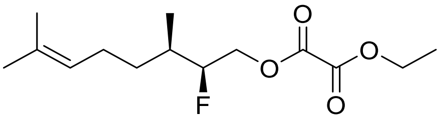

^{1}H NMR (400 MHz, Chloroform-*d*)

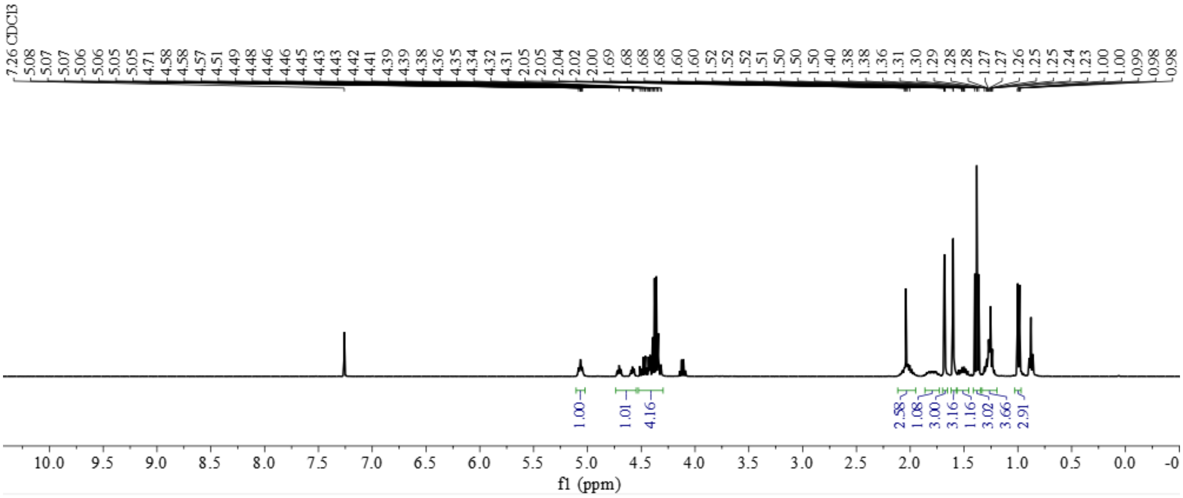

^{13}C NMR (101 MHz, Chloroform-*d*)

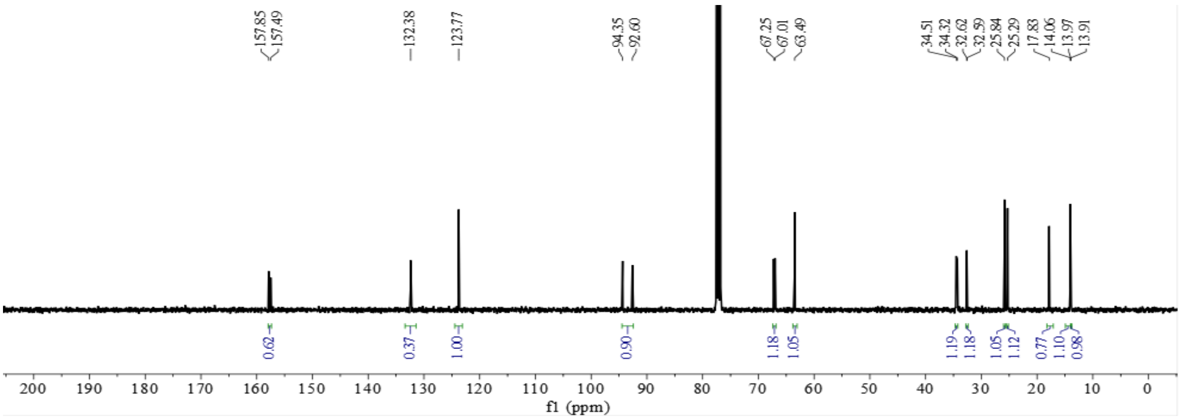

^{19}F NMR (377 MHz, Chloroform-*d*)

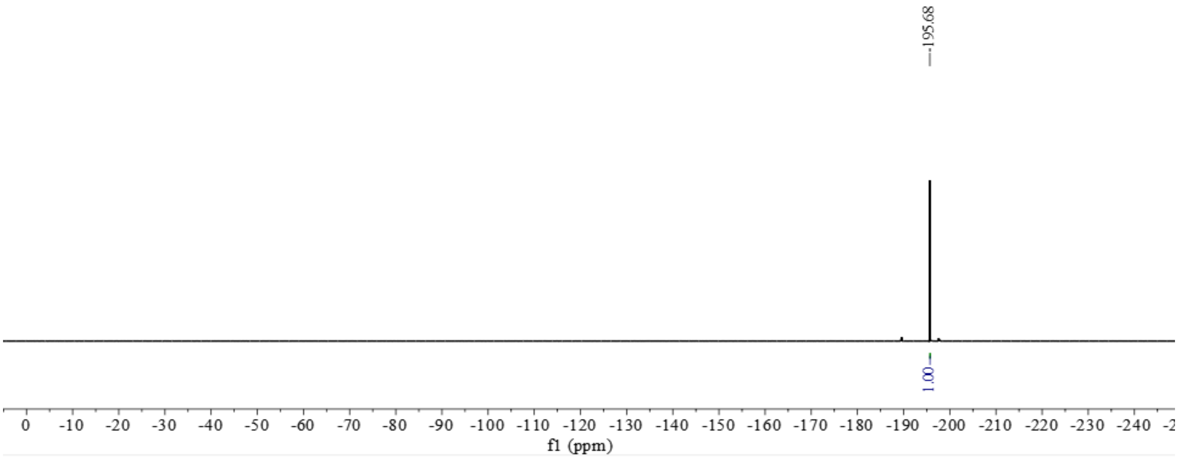

Ethyl ((2*R*,3*R*)-2-fluoro-3,7-dimethyloct-6-en-1-yl) oxalate (6)

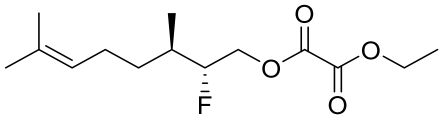

^{1}H NMR (500 MHz, Chloroform-*d*)

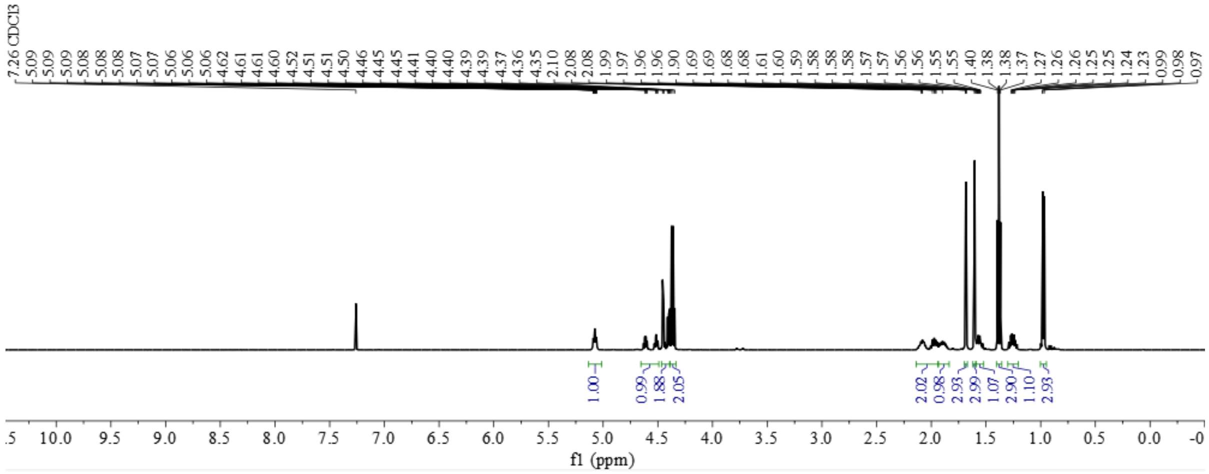

^{13}C NMR (101 MHz, Chloroform-*d*)

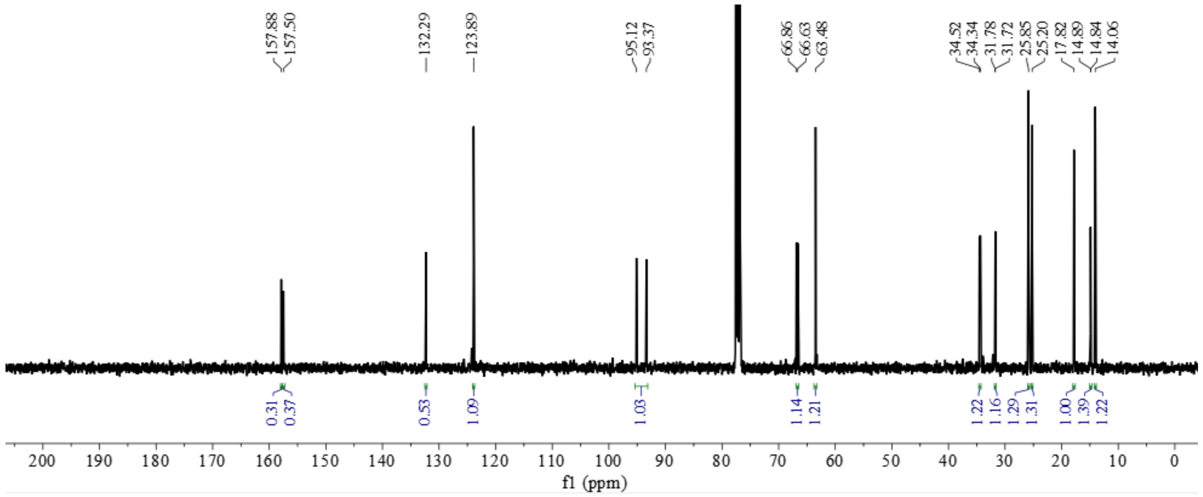

^{19}F NMR (470 MHz, Chloroform-*d*)

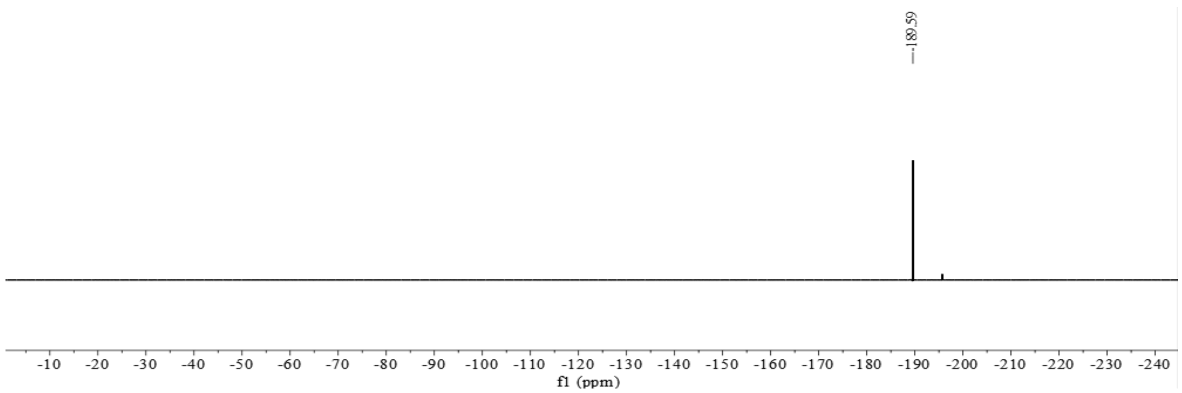

^{1}H-^{1}H-COSY (500 MHz, Chloroform-*d*)

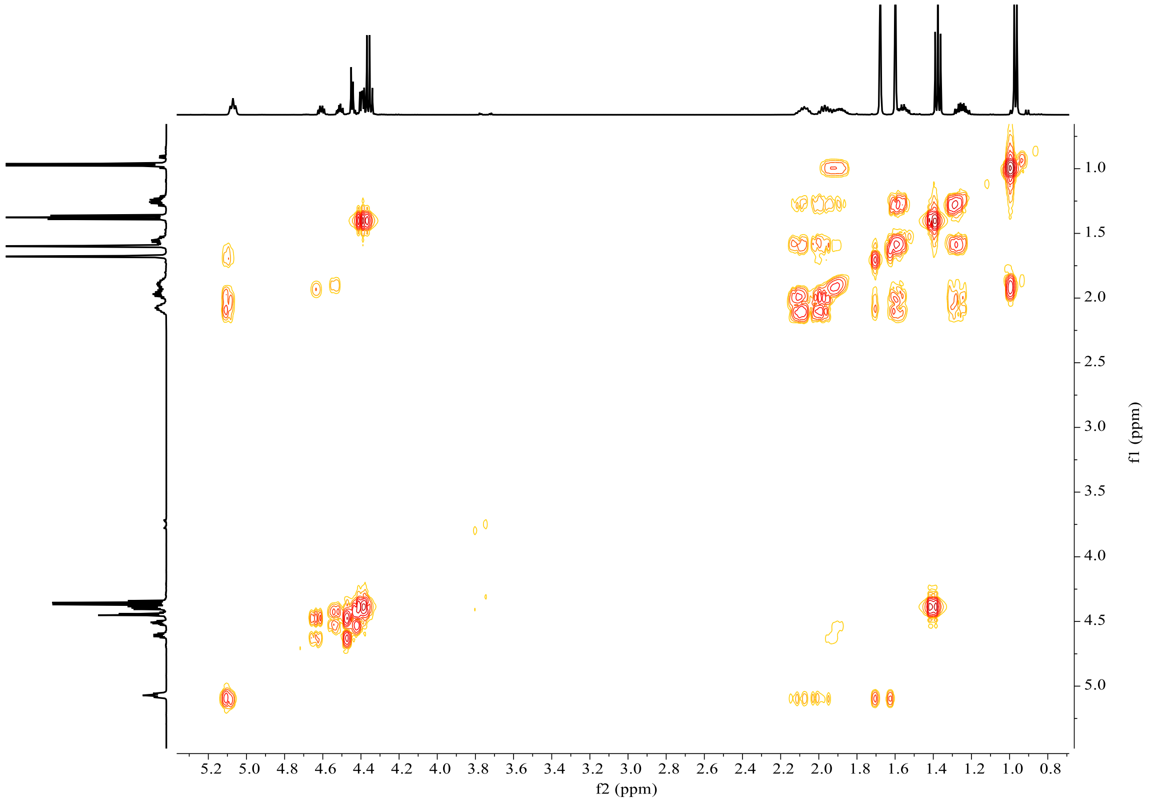

^{1}H-^{13}C-HSQC (500 MHz and 101 MHz, Chloroform-*d*)

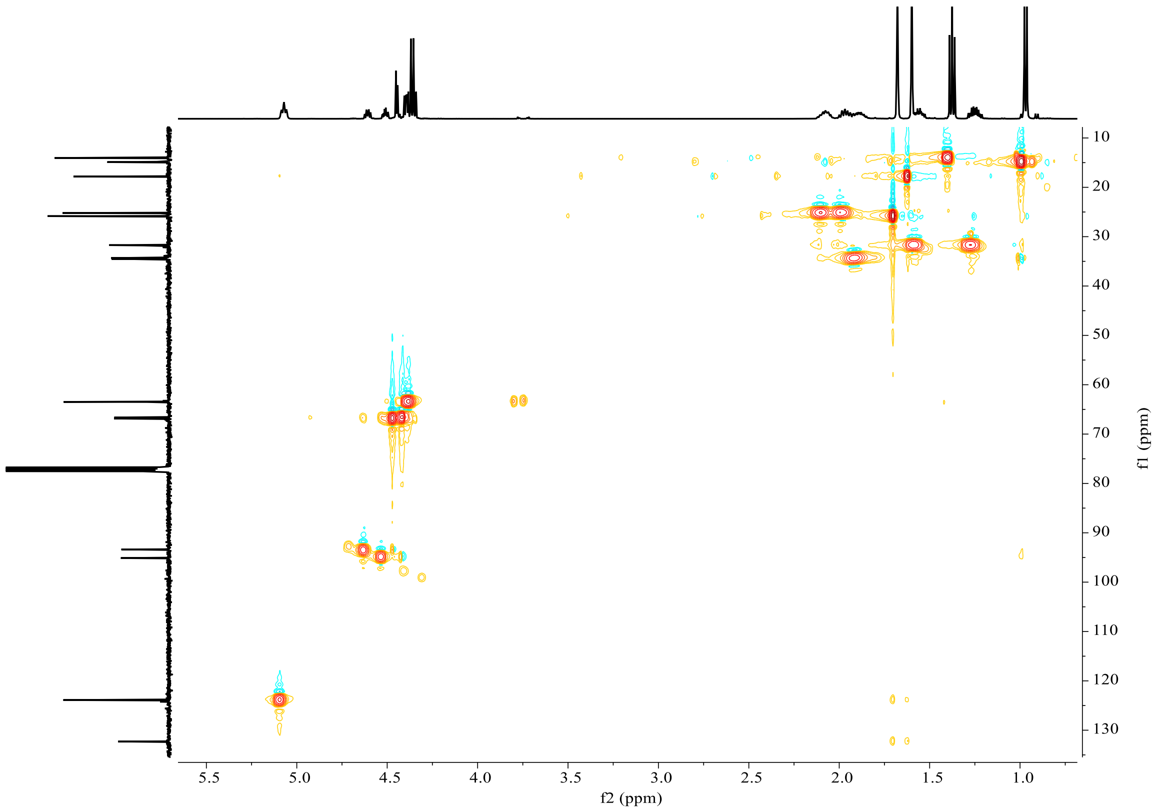

^1H-^{13}C-HMBC (500 MHz and 101 MHz, Chloroform-*d*)

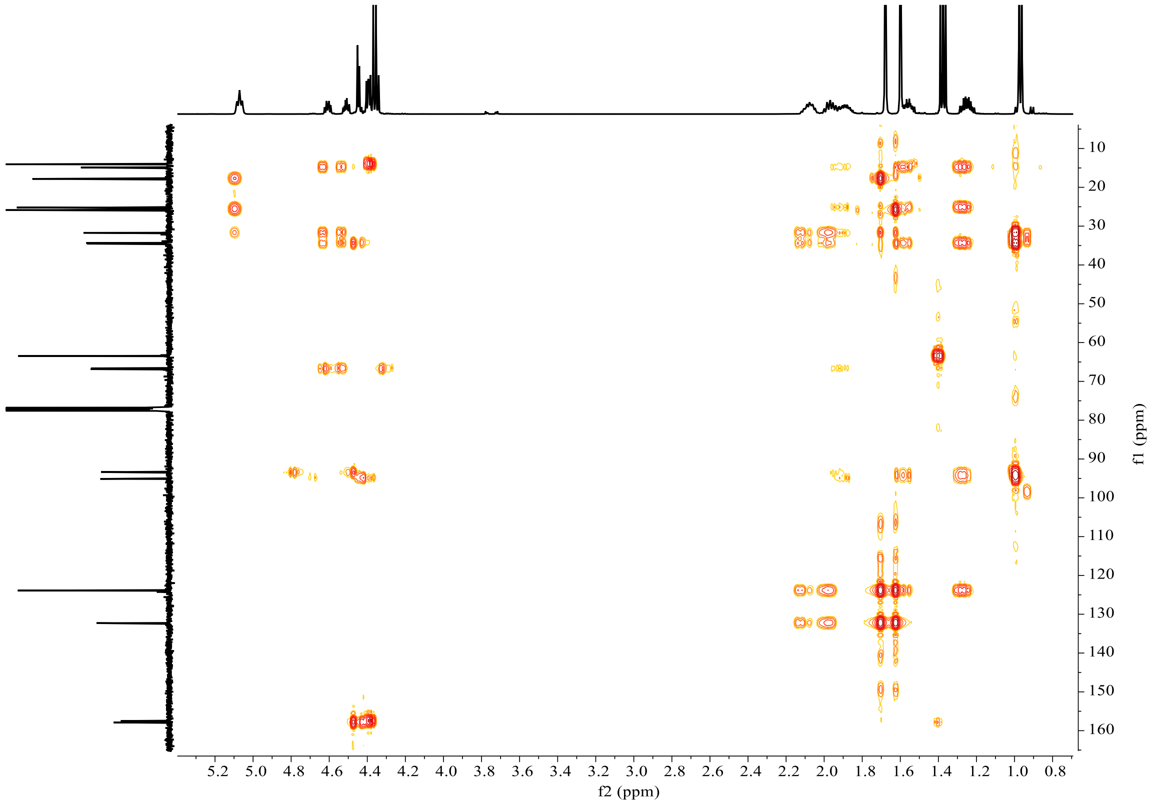

Ethyl ((2*S*,3*R*)-2,3,7-trimethyloct-6-en-1-yl) oxalate (9)

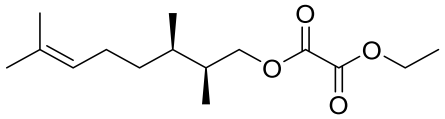

^{1}H NMR (300 MHz, Chloroform-*d*)

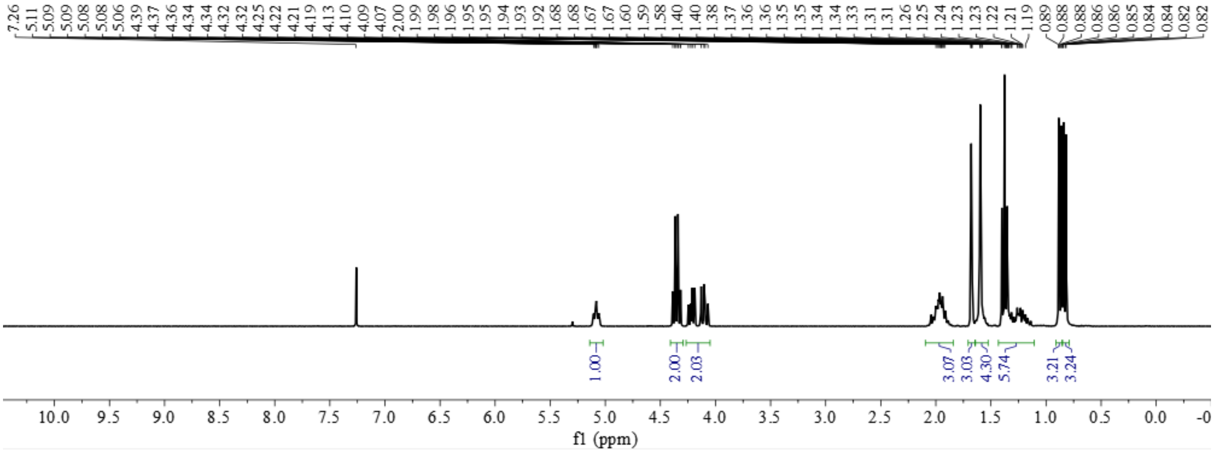

^{13}C NMR (101 MHz, Chloroform-*d*)

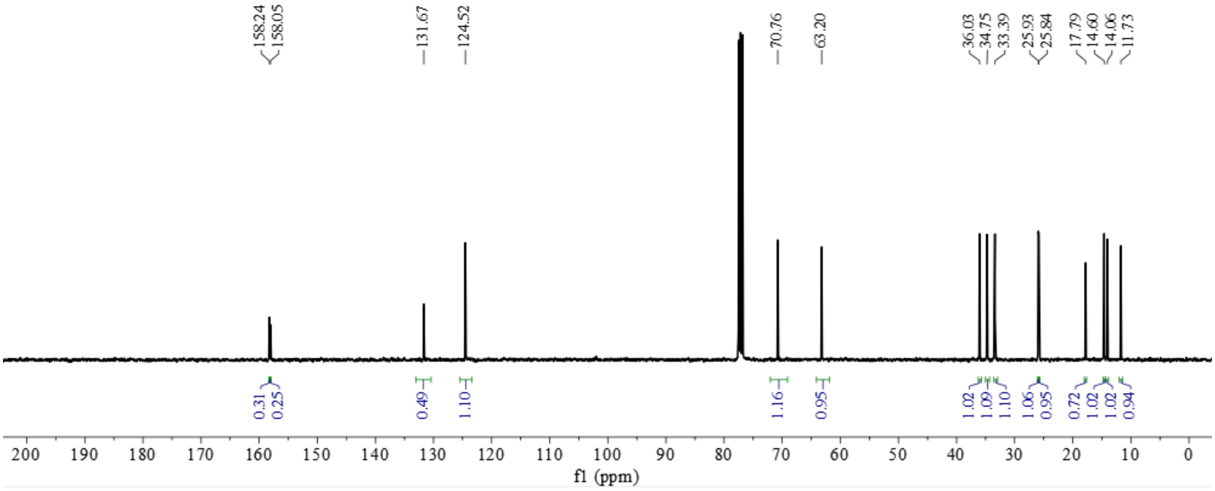

^{1}H-NOESY (700 MHz, Chloroform-*d*)

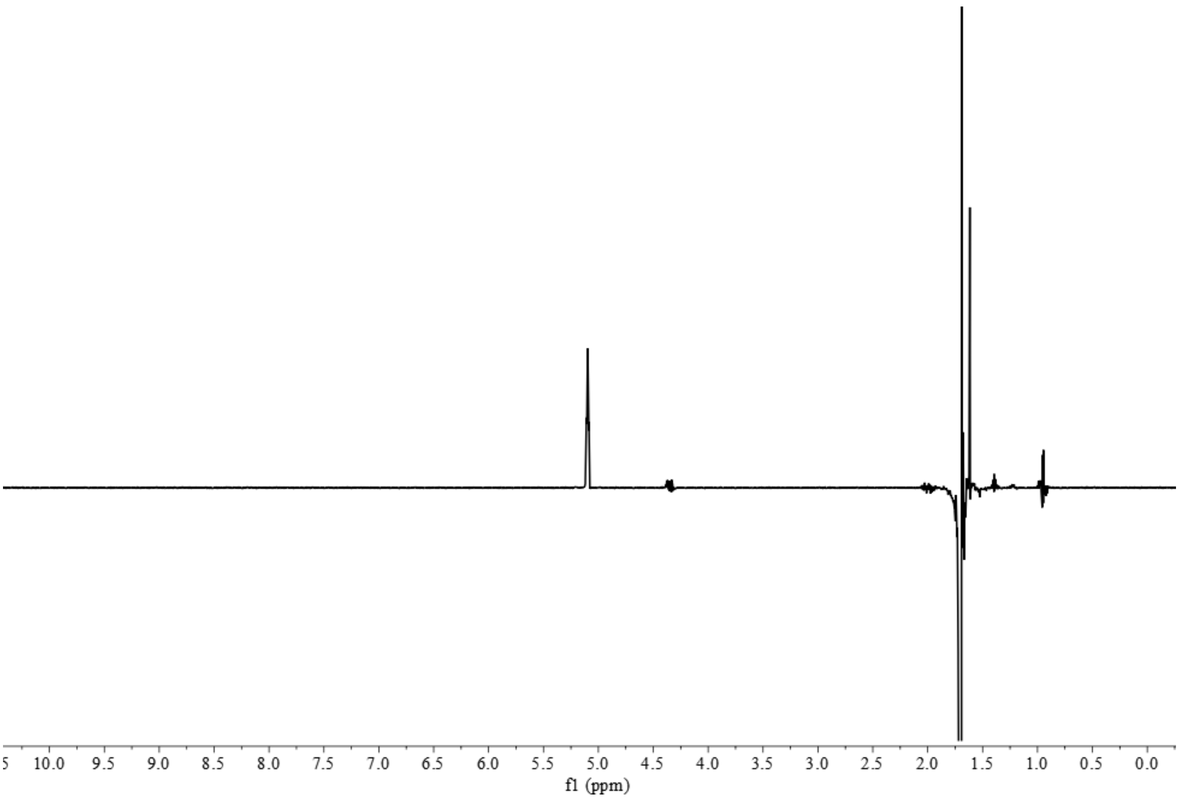

^{1}H-^{1}H-COSY (300 MHz, Chloroform-*d*)

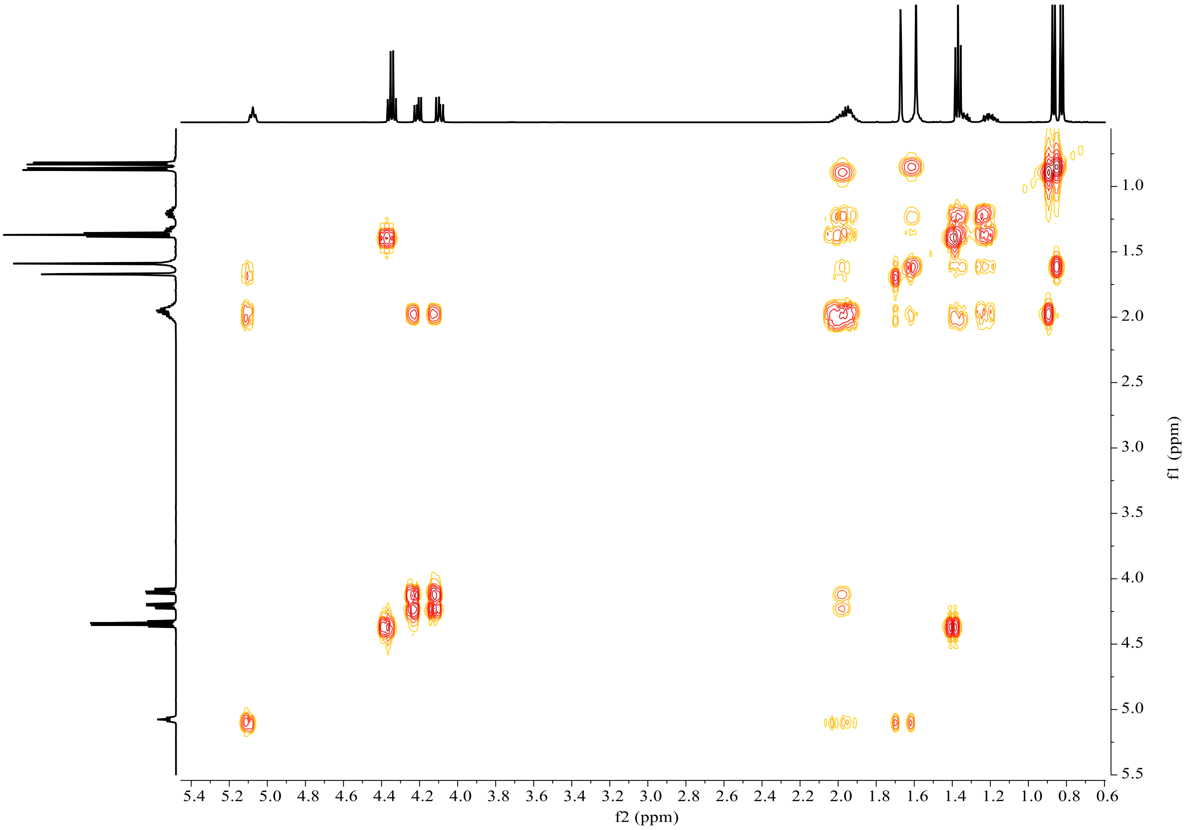

^{1}H-^{13}C-HSQC (300 MHz and 101 MHz, Chloroform-*d*)

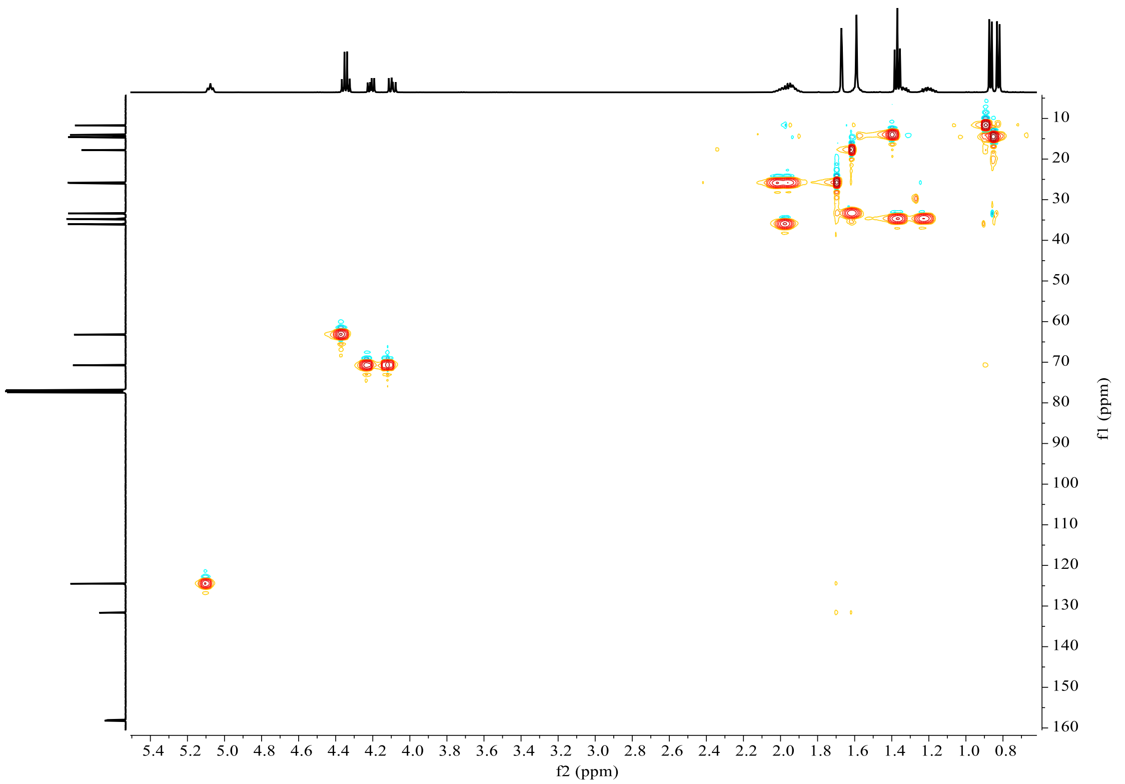

^{1}H-^{13}C-HMBC (300 MHz and 101 MHz, Chloroform-*d*)

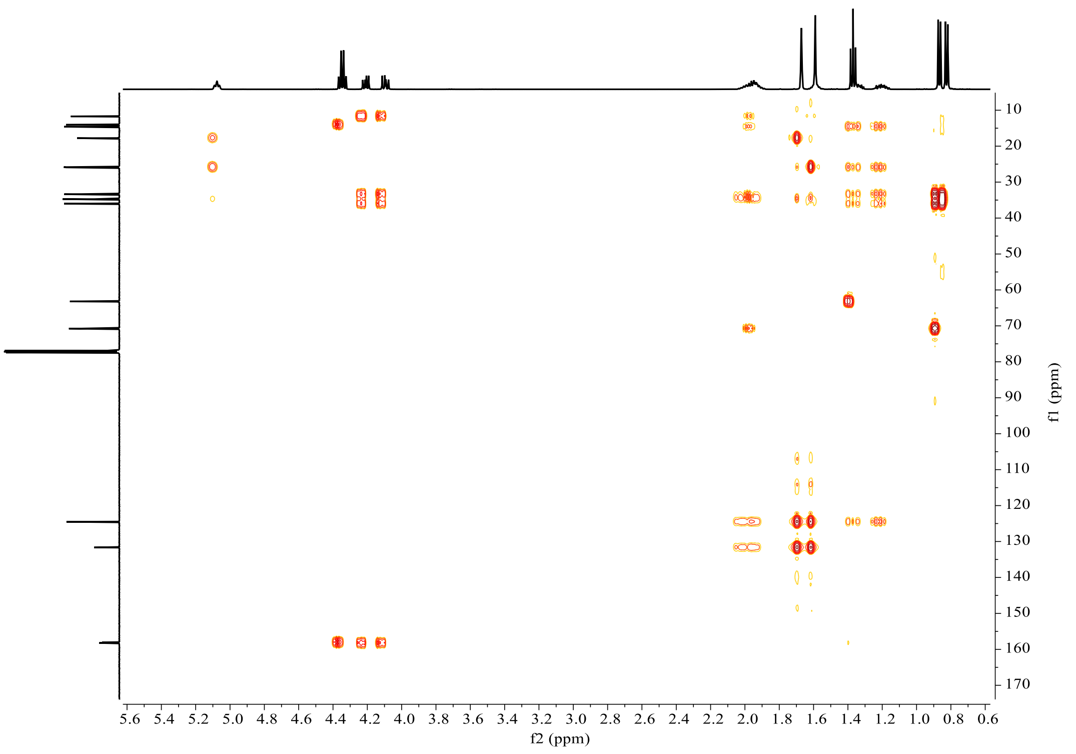

Ethyl ((2*R*,3*R*)-2,3,7-trimethyloct-6-en-1-yl) oxalate (10)

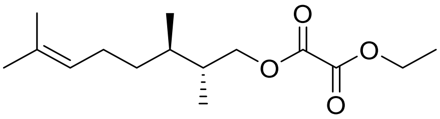

^{1}H NMR (300 MHz, Chloroform-*d*)

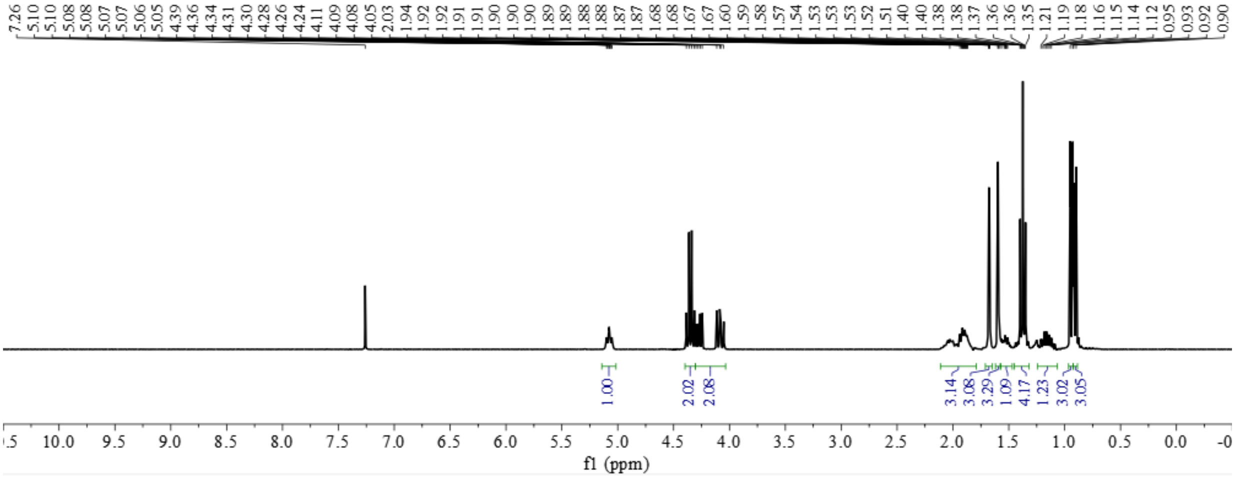

^{13}C NMR (101 MHz, Chloroform-*d*)

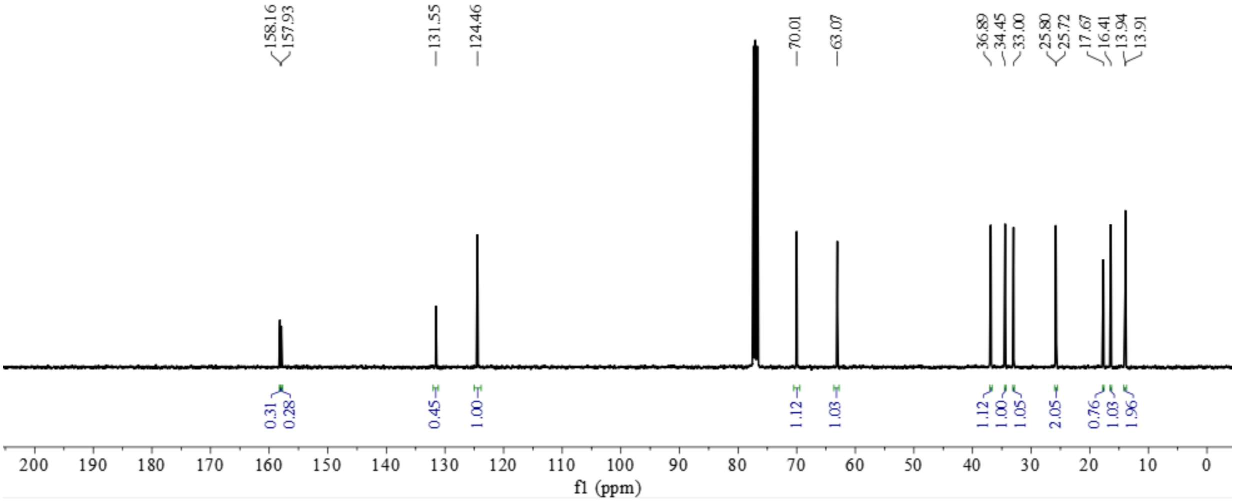

^{1}H-NOESY (700 MHz, Chloroform-*d*)

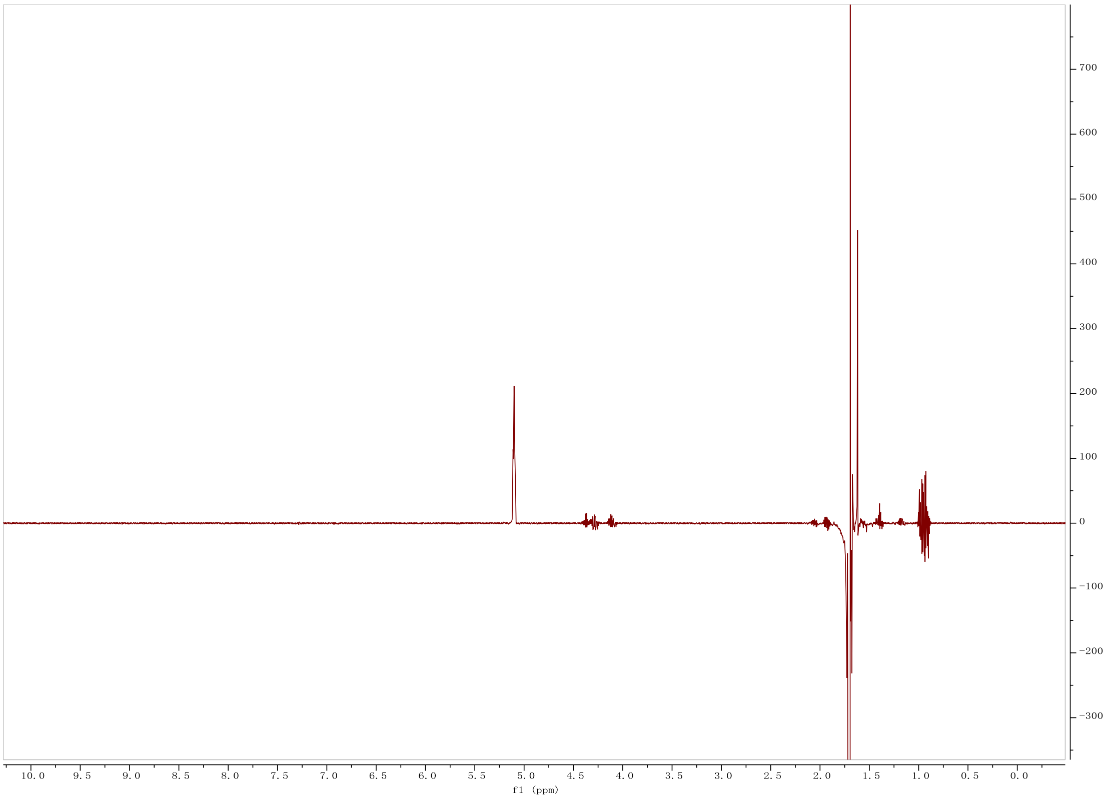

^{1}H-^{1}H-COSY (300 MHz, Chloroform-*d*)

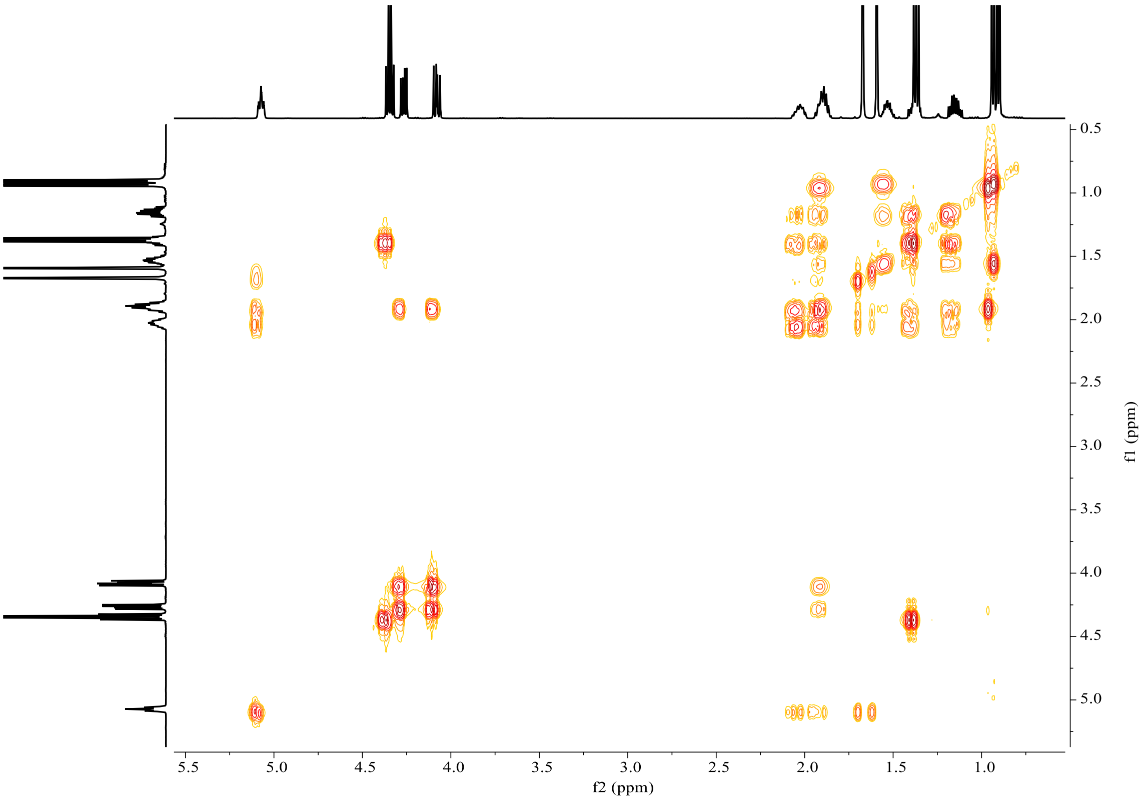

^{1}H-^{13}C-HSQC (300 MHz and 101 MHz, Chloroform-*d*)

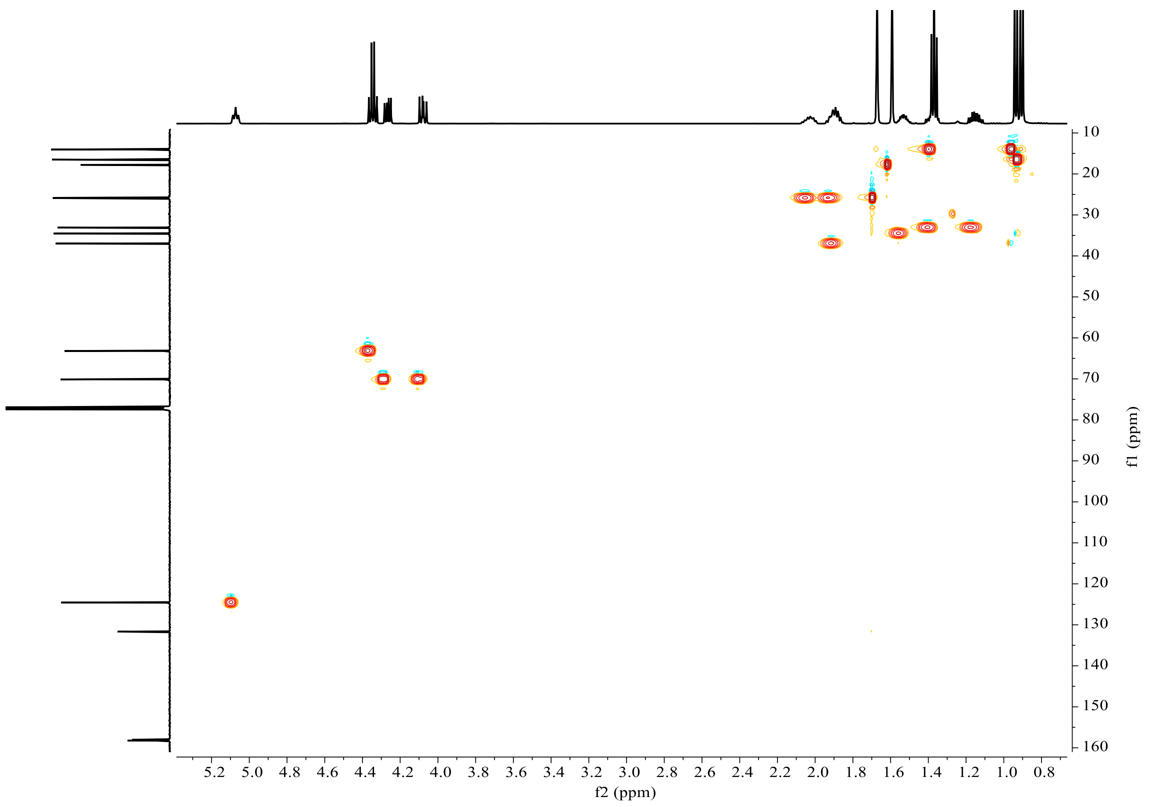

^{1}H-^{13}C-HMBC (300 MHz and 101 MHz, Chloroform-*d*)

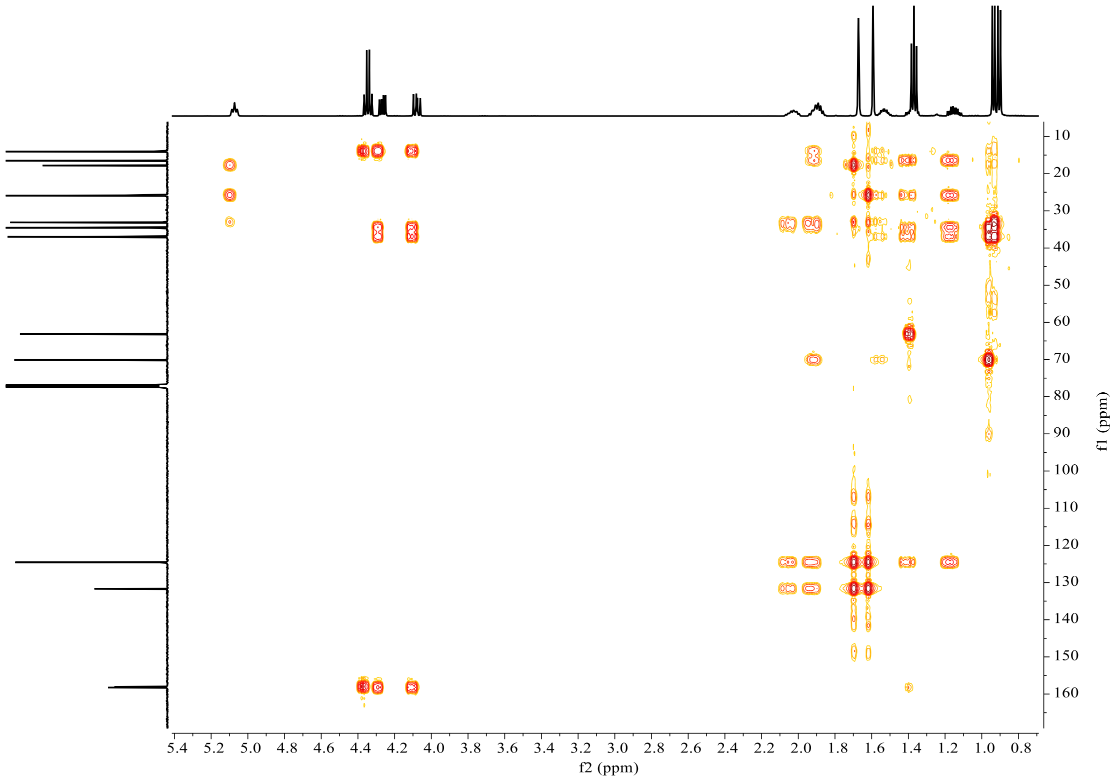

Additional experimental procedures

Clones and Mutagenesis

OR1A1 and an N-terminal rhodopsin tag were cloned into the pCI mammalian expression vector, as described previously.[8] For OR1A1 active site mutants, site-directed mutagenesis was carried out using overlap extension PCR. The identities of all constructs were confirmed by sequencing.

Luciferase assay

HEK293T-derived Hana3A cell line was grown in minimum essential medium containing 10% FBS at 37 °C with 5% CO_2 and were plated onto 96-well plates (Corning) for experiments. After 18–24 h, OR1A1, mRTP1S, CRE-Luciferase, and pRL-SV40 were transiently transfected into cells using Lipofectamine 2000 (Invitrogen). Twenty-four hours after transfection, the cells were stimulated with odorants dissolved in CD293 medium (Gibco). We used the Dual-Glo Luciferase kit (Promega) and followed the manufacturer's protocol for measuring chemiluminescence using a Synergy H1 plate reader (BioTek). All responses were normalised to the activity of wild type OR1A1 to (2*S*,3*R*)-monofluoro-citronellol **3**. The results were analysed with GraphPad Prism 8.

Table S1. EC_{50} values for alcohol compounds shown in Figure 2(a)

Compound	EC_{50} (μM)
**3**	551
**8**	238
**4**	327
**1**	81.4
**7**	No response
**18**	65.2

Figure S1 Oxalate ester dose response curves against the human olfactory receptor OR1A1.

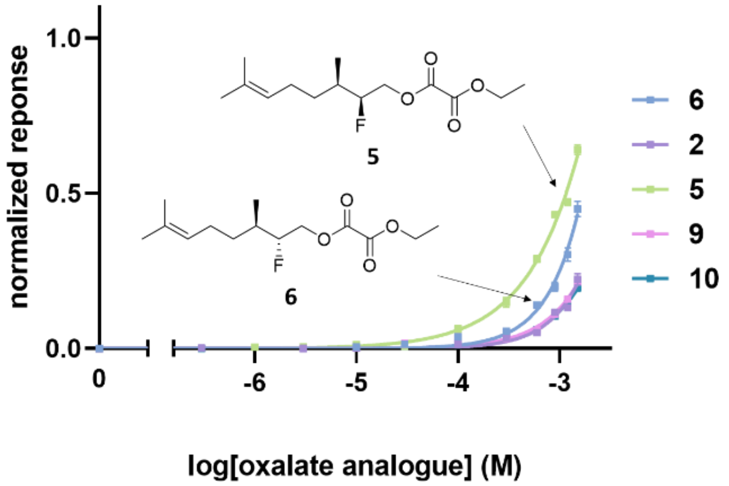

References

1. Plesek, J., Selfcondensation of cyclopentane. *Chem. Listy* **1956,** *50*, 1854.
2. (a) Overberger, C. G.; Kaye, H., Syntheses of some optically active. epsilon.-caprolactones. *J. Am. Chem. Soc.* **1967,** *89*, 5640-5645. (b) Heretsch, P.; Rabe, S.; Giannis, A., Synthesis of All Diastereomers of the Piperidine− Alkaloid Substructure of Cyclopamine. *Org. lett.* **2009,** *11*, 5410-5412.
3. (a) Chen, C. Y.; Nagumo, S.; Akita, H., A synthesis of (2R, 4'R, 8'R)-α-tocopherol (vitamin E) side chain. *Chem. Pharm. Bull.* **1996,** *44*, 2153-2156. (b) Barton, D.; Morgan, L., 117. Photochemical transformations. Part XII. The photolysis of azides. *J. Chem. Soc.* **1962**, 622-631. (c) Lee, Y. R.; Xia, L., Efficient one-pot synthetic approaches for cannabinoid analogues and their application to biologically interesting (−)-hexahydrocannabinol and (+)-hexahydrocannabinol. *Tetrahedron Lett.* **2008,** *49*, 3283-3287.
4. Grošelj, U.; Beck, A.; Schweizer, W. B.; Seebach, D., Preparation and Structures of 2-Substituted 5-Benzyl-3-methylimidazolidin-4-one-Derived Iminium Salts, Reactive Intermediates in Organocatalytic Transformations Involving α, β-Unsaturated Aldehydes. *Helv. Chim. Acta* **2014,** *97*, 751-796.
5. Abas, H.; Mas-Roselló, J.; Amer, M. M.; Durand, D. J.; Groleau, R. R.; Fey, N.; Clayden, J., Asymmetric and Geometry-Selective α-Alkenylation of α-Amino Acids. *Angew. Chem. Int. Ed.* **2019,** *58*, 2418-2422.
6. Tsuchikawa, H.; Minamino, K.; Hayashi, S.; Murata, M., Efficient Access to the Functionalized Bicyclic Pharmacophore of Spirolide C by Using a Selective Diels–Alder Reaction. *Asian J. Org. Chem.* **2017,** *6*, 1322-1327.
7. (a) Yang, H.-J.; Wang, W., Solubility comparison of bis (2-hydroxyethyl) ether and tetraethylene glycol before and after end-group modification by ethyl oxalyl chloride in supercritical carbon dioxide. *J. Chem. Eng. Data.* **2010,** *55*, 2279-2283. (b) Yamamoto, T.; Shimada, A.; Ohmoto, T.; Matsuda, H.; Ogura, M.; Kanisawa, T., Olfactory study on optically active citronellyl derivatives. *Flavour frag. J.* **2004,** *19*, 121-133.
8. Saito, H.; Kubota, M.; Roberts, R. W.; Chi, Q.; Matsunami, H., RTP family members induce functional expression of mammalian odorant receptors. *Cell* **2004**, *119*, 679–691.
